# Sulfur(iv) reagents for the SuFEx-based synthesis of substituted sulfamate esters†

**DOI:** 10.1039/d2sc05945b

**Published:** 2023-01-20

**Authors:** Kathleen T. Downey, Jia Yi Mo, Joey Lai, Brodie J. Thomson, Glenn M. Sammis

**Affiliations:** a Department of Chemistry, The University of British Columbia 2036 Main Mall Vancouver British Columbia V6T 1Z1 Canada gsammis@chem.ubc.ca

## Abstract

Sulfur(vi) fluoride exchange chemistry has been reported to be effective at synthesizing valuable sulfur(vi) functionalities through sequential nucleophilic additions, yet oxygen-based nucleophiles are limited in this approach to phenolic derivatives. Herein, we report a new sulfur(iv) fluoride exchange strategy to access synthetically challenging substituted sulfamate esters from alkyl alcohols and amines. We also report the development of a non-gaseous, sulfur(iv) fluoride exchange reagent, *N*-methylimidazolium sulfinyl fluoride hexafluorophosphate (MISF). By leveraging the reactivity of the sulfur(iv) center of this novel reagent, the sequential addition of alcohols and amines to MISF followed by oxidation afforded the desired substituted sulfamates in 40–83% yields after two steps. This new strategy expands the scope of SuFEx chemistry by increasing the accessibility of underdeveloped –S(O)F intermediates for future explorations.

## Abstract 2

*N*-Methylimidazolium sulfinyl fluoride hexafluorophosphate (MISF) was developed as a solution-stable sulfur(iv) reagent to access substituted sulfamate esters using a sulfur(iv) fluoride exchange strategy.

## Graphical abstract

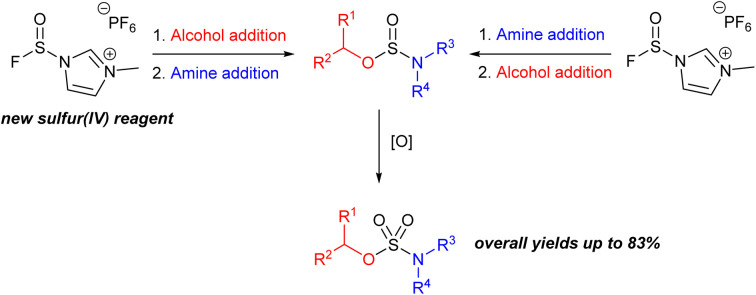

Sulfur fluoride exchange (SuFEx) reagents have powerful applications in pharmaceuticals, chemical biology, and materials science.^1^ The most commonly utilized reagents are sulfur(vi) compounds, such as sulfuryl fluoride (SO_2_F_2_) and its derivatives, that can be used to efficiently synthesize a wide range of functionalities through sequential nucleophilic additions (Fig. 1a).^2^ Sulfamate esters are targets of particular interest as they display potential as anticancer agents and as a new class of antibiotics,^3^ as well as versatility as synthetic intermediates.^4^ Oxygen-based nucleophiles are limited to phenolic derivatives with sulfur(vi) SuFEx reagents, restricting their use in sulfamate ester syntheses. The reaction of aliphatic alcohols with SO_2_F_2_ leads to aliphatic fluorosulfate intermediates that are very unstable, and results in rapid substitution at the fluorosulfate alpha-position (Fig. 1b).^5,6^*O*-Alkyl sulfamate esters (ROSO_2_NH_2_) are readily synthesized with alternatives to SuFEx-based methods,^7^ but the substituted analogues require harsh conditions, multiple steps, and long reaction times.^8^ For example, sulfur(vi) chloride reagents have been utilized in the syntheses of sulfur(vi) moieties, yet are limited due to their inherent instability and prevalent side reactions.^9,10^ A fast, mild, and SuFEx-based approach to the syntheses of substituted alkyl sulfur(vi) motifs is, therefore, desired.

**Fig. 1:**
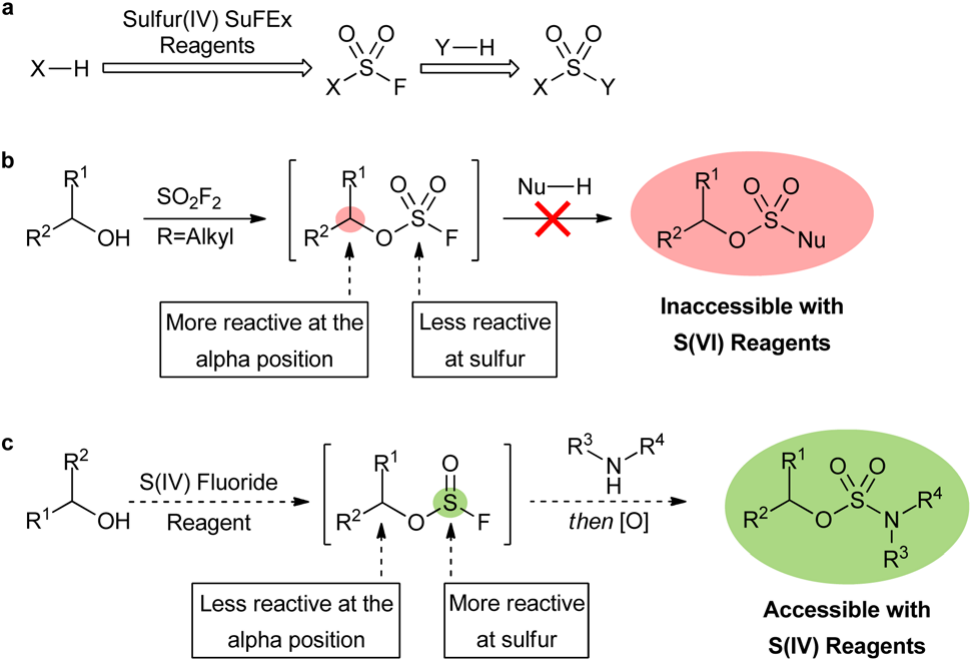
SuFEx chemistry for the syntheses of S(vi) functionalities. (a) Reported sulfur(vi) SuFEx approach to linking nucleophiles. (b) Limitations of sulfur(vi) reagents in accessing fluorosulfate intermediates. (c) This work. Use of novel sulfur(iv) SuFEx reagent followed by oxidation to access synthetically challenging sulfur(vi) motifs that are inaccessible with sulfur(vi) reagents.

The aforementioned limitations of sulfur(vi) SuFEx reactions may be overcome by utilizing a novel approach involving sulfur(iv) fluoride reagents (Fig. 1d). The addition of an alkyl alcohol to a sulfur(iv) fluoride exchange reagent should readily form the corresponding fluorosulfite intermediate. In contrast to fluorosulfates, fluorosulfites 2 may be more reactive at the sulfur center than the alpha-position.^11^ Therefore, the addition of a heteroatom nucleophile to the sulfur(iv) center of a fluorosulfite should be faster and more selective than the sulfur(vi) analogue. A subsequent oxidation of the sulfur(iv) center would then afford the desired sulfur(vi) motif. While the oxidation step limits the substrate scope, this strategy provides a route to substrates that were previously inaccessible.

Thionyl fluoride (SOF_2_), the sulfur(iv) analogue of SO_2_F_2_, has displayed versatile reactivity despite the relatively few studies on its use.^11,12^ The initial addition of a heteroatomic nucleophile to thionyl fluoride has been reported to be an efficient protocol for achieving amino sulfinyl fluorides and fluorosulfites,^13^ yet further reactivity of these intermediates has not been reported. There are also several major drawbacks to its use, including safety hazards associated with handling the gaseous reagent.^14^ There is no literature precedence for non-gaseous sulfur(iv) derivatives of thionyl fluoride. Therefore, a non-gaseous derivative that maintains the fundamental reactivity of SOF_2_ is essential for the broad adoption of this class of sulfur(iv) reagents.

## Results and discussion

### Reagent development

Our investigations initially focused on developing new, non-gaseous S(iv) derivatives of thionyl fluoride. Once synthesized, the reactivity of our new S(iv) reagents was examined in the conversion of 3-phenylpropanol to the corresponding fluorosulfite (Table 1).^15^ While we were unable to prepare the S(iv) analogue of [4-(acetylamino)phenyl]imidodisulfuryl difluoride (AISF),^16^ a mixed S(vi)–S(iv) analogue (3) was synthesized. Unfortunately, 3 did not convert 1a to 2a. We next prepared thionyl diimidazole (TDI, 4),^17^ but no fluorosulfite was observed when 1a was treated with 1 equivalent of both TDI and KF. Further optimization with TDI was not investigated as it is unstable and readily decomposed both as a solid and in solution.^18^ While 1,2-dimethylimidazole sulfinyl fluoride 5 converted 1a to 2a in 17% yield, *N*-methylbenzimidazole sulfinyl fluoride 6 was unsuccessful. *N*-Benzylimidazolium derivative 7 showed greater success in the formation of 2a, albeit in only 60% yield. Gratifyingly, *N*-methylimidazolium sulfinyl fluoride hexafluorophosphate (MISF) led to 76% yield of fluorosulfite 2a in 15 minutes. Addition of one equivalent of KF and MISF promoted the formation of fluorosulfite 2a in quantitative yield.^19^ Treatment of 3-phenylpropanol with *N*′,*N*-dimethylimidazoliumsulfoxide hexafluorophosphate (DIMISF) and KF afforded 2a in 80% yield. DIMISF (9) displayed similar stability in solution and decomposition upon isolation. As a result, we proceeded forward with MISF as it indicated improved yields and better atom economy.

**Table 1:** Development of S(iv) reagent based on reported S(vi) compounds^a^

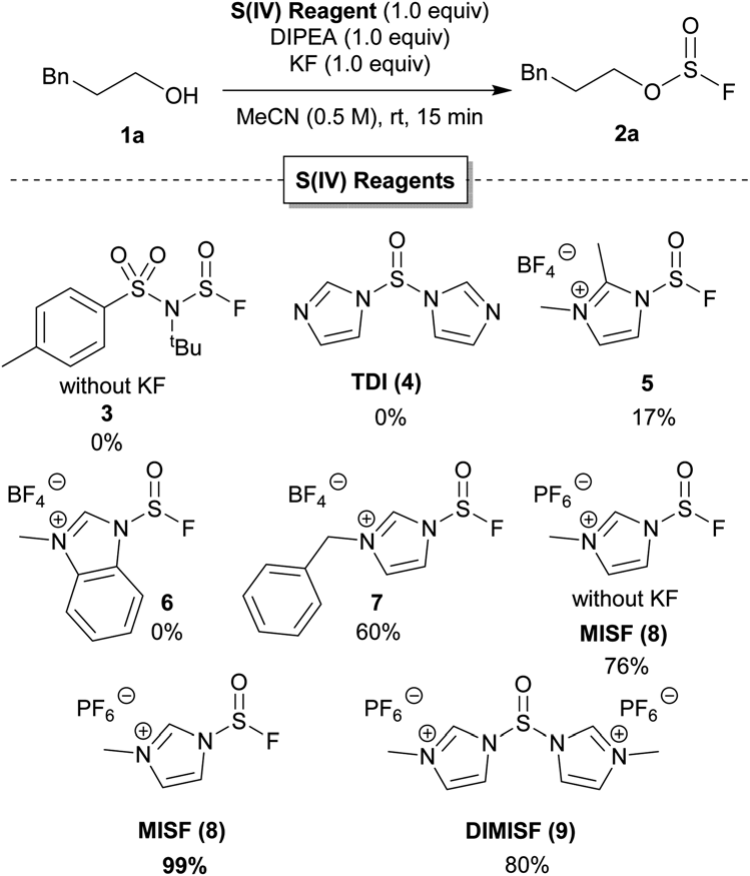

a Representative S(iv) reagents tested as possible deoxyfluorination agents to afford 3-phenylpropyl sulfurofluoridite from 3-phenylpropanol. Reactions were performed on 0.6 mmol scale. Yields were determined by ^19^F NMR spectroscopy with trifluorotoluene as an internal standard.

We next assessed the stability of MISF in solution and as a solid. MISF was stored as a 0.19 M solution in acetonitrile with no observable decomposition by ^1^H NMR spectroscopy over a period of three months under either inert atmosphere or air.^20^ In contrast, the concentration of thionyl fluoride in stock solutions decreases notably over similar timeframes, limiting its use as a solution-based reagent.^21^ At the end of the third month, MISF stored under both argon and air each afforded quantitative yields of 3-phenylpropyl sulfurofluoridite (2a). While MISF could not be generated effectively in diethyl ether, dichloromethane, or dioxane,^22^ solutions of MISF in tetrahydrofuran successfully promoted the conversion of 1a to 2a in 96% yield. Attempts to isolate MISF as a solid were unsuccessful as it readily decomposes during isolation or during aqueous workup, resulting in the recovery of *N*-methylimidazole and hexafluorophosphate salts.^23^

### New sulfur(iv) fluoride strategy for the synthesis of sulfamate esters

**Scheme 1:**
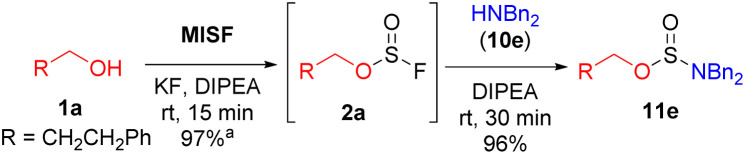
MISF-mediated sulfamidite formation. Reaction was performed at 0.6 mmol scale, MISF in MeCN (0.24 M). ^a^The yield was determined by ^19^F NMR spectroscopy using trifluorotoluene as an internal standard.

We next examined the use of MISF as the key reagent in a new method to synthesize difficult-to-access di- and tri-substituted sulfamate esters. We began our investigation into the synthesis of sulfamate esters by treating 3-phenylpropanol (1a) with MISF (1 equiv.), KF (1 equiv.) and DIPEA (1 equiv.) to afford the corresponding alkyl fluorosulfite in quantitative yields at room temperature in 15 minutes. As expected, the fluorosulfite intermediate was found to decompose during isolation. However, upon filtration of the reaction mixture and then treatment of the fluorosulfite with dibenzylamine (10e), the corresponding sulfamidite (11e) was isolated in 96% yield without the need for column chromatography (Scheme 1). Sulfamidite 11e required storage under anhydrous conditions as it hydrolyzed upon prolonged exposure to moisture. The same transformation was performed with thionyl chloride, yet the chlorosulfite intermediates were unstable and none of the desired sulfamidite was formed. Treatment of 1a with sulfuryl fluoride led to none of the desired fluorosulfate, and only nucleophilic substitution products were observed.^5*c*^

The oxidation of sulfamidite 11e was initially attempted using a mixture of 2,2,6,6-tetramethylpiperidinyloxy (TEMPO) and sodium hypochlorite (NaOCl). These conditions were unsuccessful, affording only the hydrolyzed products (Table 2, entry 1). While both Dess–Martin periodinane (DMP) and Oxone® were ineffective (entries 2 and 3), oxidation with 3-chloroperbenzoic acid (*m*CPBA) led to a 20% conversion to sulfamate 12e (entry 4). A mixture of potassium permanganate (KMnO_4_) and manganese dioxide (MnO_2_) further increased the conversion of 12e to 60% (entry 5). Ruthenium tetroxide (RuO_4_), generated *in situ* from ruthenium(iii) chloride (RuCl_3_) and sodium periodate (NaIO_4_),^24^ afforded over 95% conversion to 12e (entry 6).

**Table 2:** Optimization of sulfamidite oxidation^a^

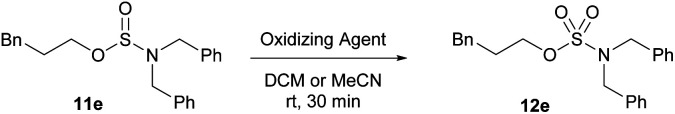		
Entry	Oxidizing agent	Conversion to 12e
1	TEMPO (10 mol%)	0%
	NaClO (1.5 equiv.)	
2	DMP (3.0 equiv.)	0%
3	Oxone® (2.0 equiv.)	0%
4	*m*CPBA (1.5 equiv.)	20%
5	MnO_2_ (2.0 equiv.)	60%
	KMnO_4_ (10 equiv.)	
**6**	**RuCl**_**3**_ **·H**_**2**_ **O (8 mol%)**	**>95%**
	**NalO**_**4**_ **(1.7 equiv.)**	

a Reactions were performed on 0.6 mmol scale. Conversion was determined by ^1^H NMR spectroscopy.

With optimized conditions in hand, the amine scope of the sulfamate ester synthesis was explored using 3-phenylpropanol as the alcohol component (Table 3). Cyclic secondary amines were effective substrates for this reaction, affording 12a, 12b, and 12c in 72%, 73%, and 54% isolated yields, respectively. *N*-Methylphenylethylamine and dibenzylamine were also able to obtain the corresponding sulfamates 12d in 79% and 12e in 83% isolated yields. Diethylamine and *N*,*O*-dimethylhydroxylamine were viable substrates with sulfamate yields of 55% and 63%, respectively. Primary amines were unsuccessful using this method as sulfoxide diamides were obtained. To overcome this limitation, PMB-protected propylamine was converted to sulfamate 12h in 60% yield and then the PMB-group was removed in quantitative yield.^25^ The protocol was also found to be tolerant of amides as sulfamate 12i was afforded in 60% isolated yield. This method was also found to be effective with oxidizable substrates, with piperazine sulfamate 12j successfully synthesized in 79% yield.

**Table 3:** Amine scope of MISF-mediated synthesis of sulfamate esters^a^

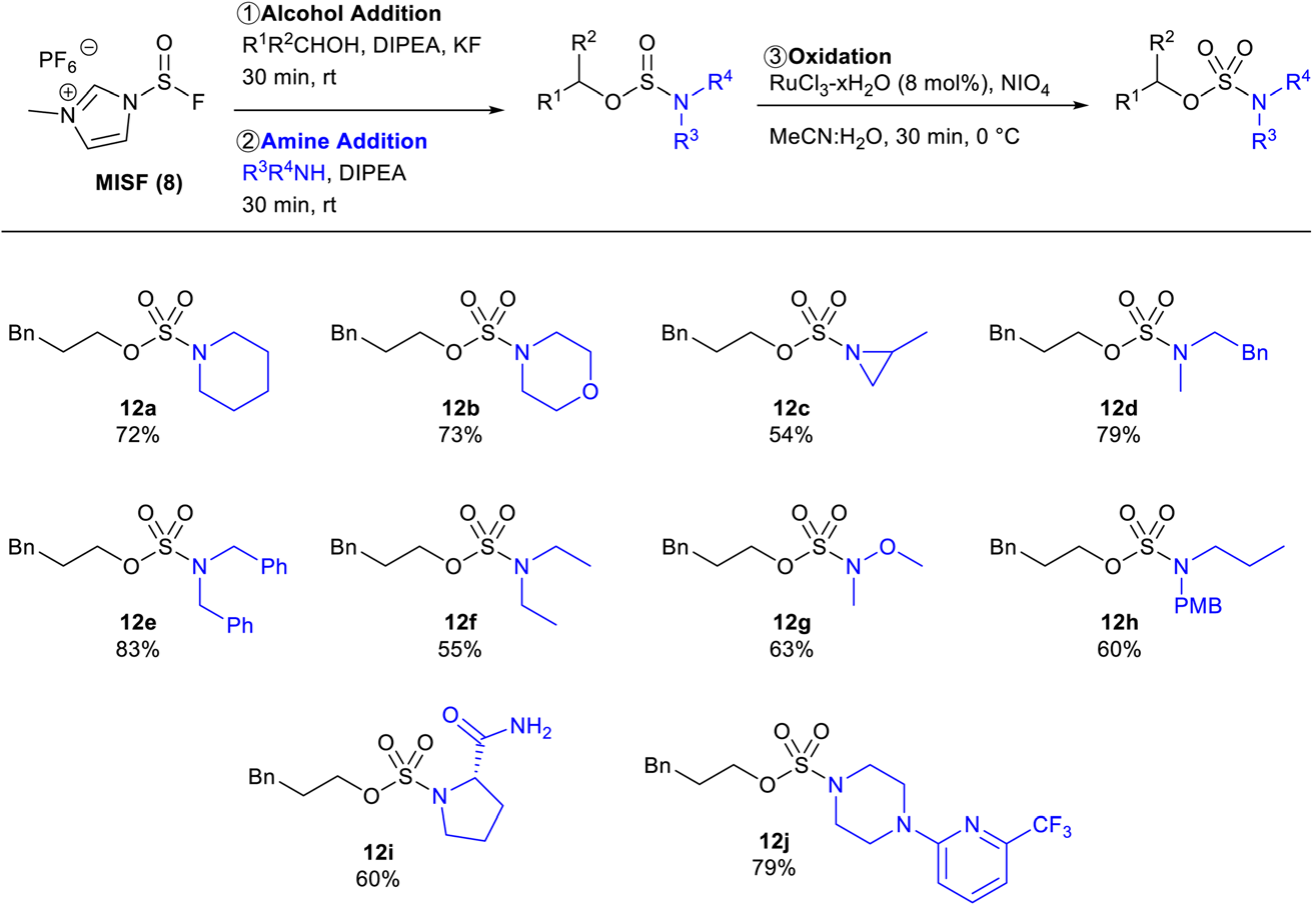

a Reactions were performed on 0.6 mmol scale.

The alcohol scope of this new sulfur(iv) SuFEx protocol was investigated using dibenzylamine as the amine components (Table 4). 4-Fluorobenzyl alcohol was an effective substrate, affording sulfamate ester 12k in 63% isolated yield over two steps. Substrates that are prone to substitution at the alpha-position with sulfur(vi) reagents (12l–12o) were successfully converted to the corresponding sulfamate esters in 79%, 63%, 78%, and 70% isolated yields, respectively. The reaction was also shown to be effective with sterically hindered substrates, as shown by the good yield of sulfamate ester 12p. 4-Pyridinemethanol was a viable substrate generating sulfamate 12q in 40% yield. The lower yield was a result of the decreased reactivity between 4-pyridinemethanol and MISF.

**Table 4:** Alcohol scope of MISF-mediated sulfamate ester synthesis^a^

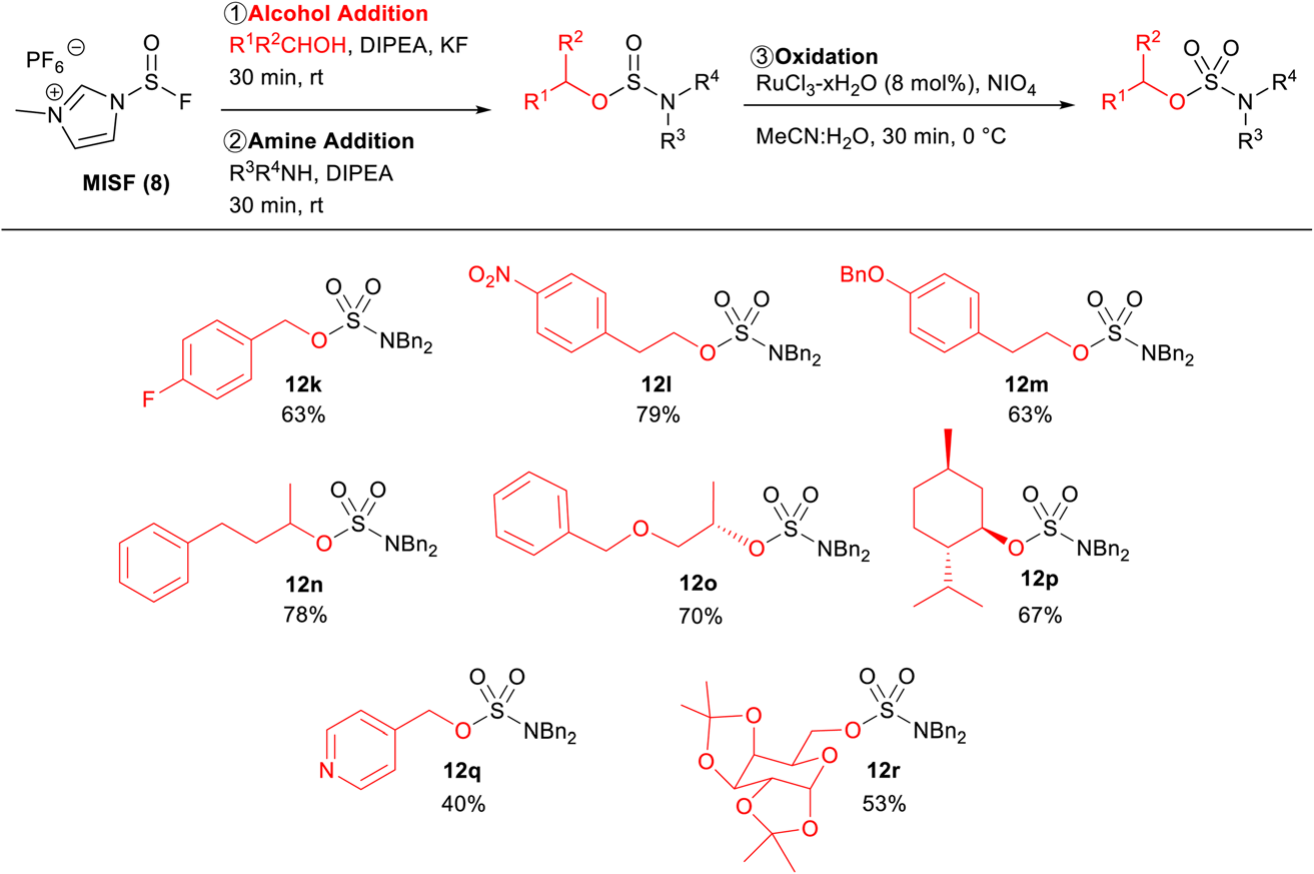

a Reactions were performed on 0.6 mmol scale.

Finally, protected monosaccharide 1r was also a successful substrate, affording sulfamate 12r in 53% isolated yield. The installation of sulfamate esters onto protected monosaccharides is of particular interest due to their desirable pharmacological activity.^3*d*^

We next explored whether we could synthesize the same sulfamate esters by reversing the order of nucleophile addition, whereby the amine is added first to afford the aminosulfinyl fluoride as the key intermediate (Table 5). The amine scope was explored with 3-phenylpropanol as the alcohol component. Cyclic secondary amines were effective substrates as sulfamate 12a was obtained in 52% yield. Acyclic secondary amines were also effective substrates affording sulfamate esters 12d, 12e, and 12h in 71%, 82%, and 57% isolated yields, respectively. The alcohol scope was also investigated using dibenzylamine as the amine component. 4-Fluorobenzyl alcohol afforded sulfamate ester 12k in 52% yield. Substrates that are prone to substitution at the alpha-position with sulfur(vi) analogues, such as sulfamates 12l, 12m, and 12n, were successfully generated with MISF in 70%, 65%, and 52% isolated yields, respectively. This reversed addition order successfully afforded the desired sulfamate esters in good yields, albeit typically lower than the strategy outlined in Tables 3 and 4. This disparity is likely the outcome of the difference in stability of the sulfinyl fluoride intermediates as it was observed that aminosulfinyl fluorides hydrolyze faster than fluorosulfites.^26^

**Table 5:** Substrate scope of reverse addition of MISF-mediated synthesis of sulfamate esters^a^

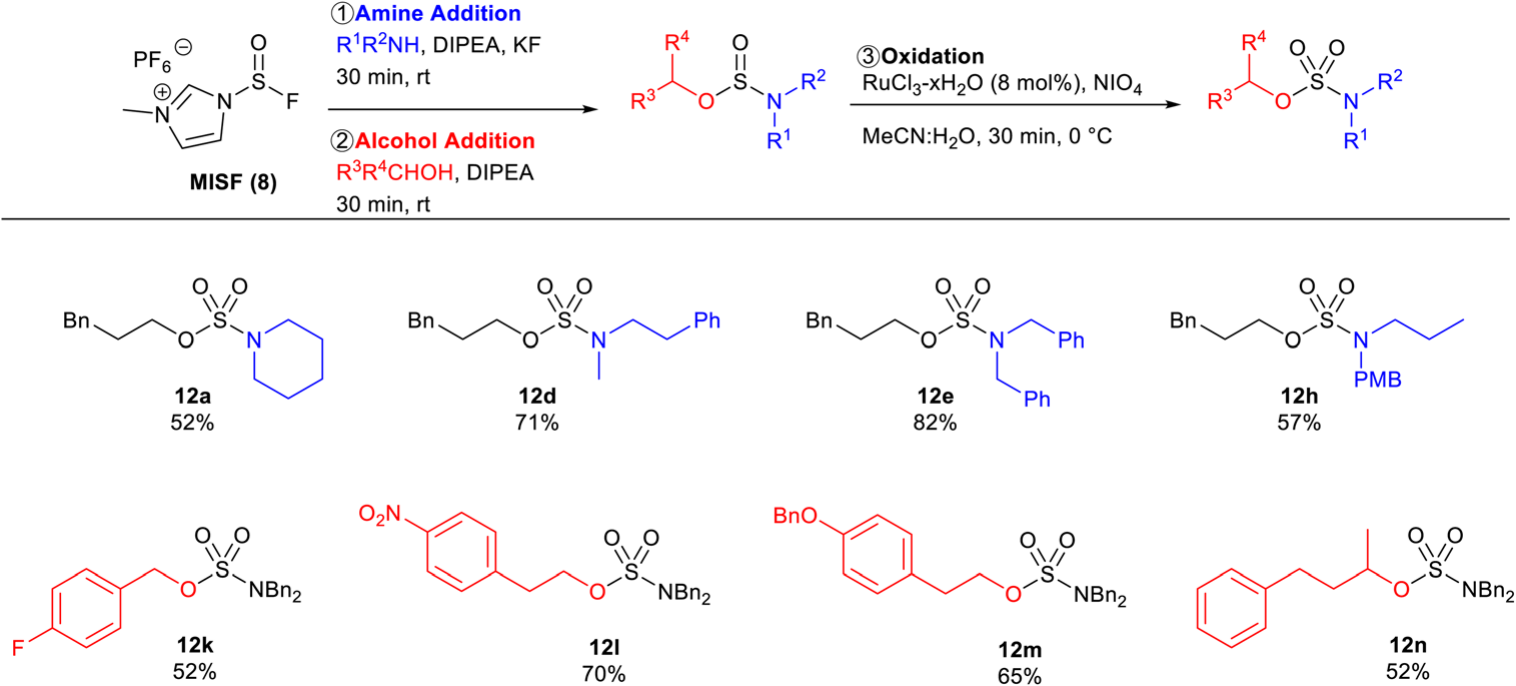

a Reactions were performed on a 0.6 mmol scale.

## Conclusions

Overall, we have developed a new SuFEx strategy using sulfur(iv) reagents to access synthetically challenging sulfamate esters. To facilitate this process, we have developed *N*-methylimidazolium sulfinyl fluoride hexafluorophosphate (MISF), a novel, solution-stable, non-gaseous sulfur(iv) SuFEx reagent. This approach affords sulfamate esters from alkyl alcohols and amines that are inaccessible using sulfur(vi) SuFEx reagents. While our process is formally two-steps, the intermediate can be purified by a simple wash, and the total reaction times are <2 hours. This novel protocol afforded the di- and tri-substituted *O*-alkyl sulfamate esters in good yields regardless of the nucleophile addition order. The stability and reactivity of MISF expands the accessibility of underexplored –S(O)F intermediates for future explorations in synthetic chemistry.

## Data availability

The datasets supporting this article have been uploaded as part of the ESI† material.

## Author contributions

K. T. D., J. Y. M., B. J. T., and G. M. S. conceived the project. K. T. D., J. Y. M., and J. L. conducted and analysed the experiments. K. T. D., J. L., B. J. T., and G. M. S. wrote the manuscript.

## Conflicts of interest

There are no conflicts to declare.

## Supplementary Material

SC-014-D2SC05945B-s001
