# Supplementary material for: Sulfur(iv) reagents for the SuFEx-based synthesis of substituted sulfamate esters
Source: Chem Sci. 2023 Jan 20;14(7):1775–80. doi: 10.1039/d2sc05945b (PMC9930924; doi:10.1039/d2sc05945b)

Electronic Supplementary Material (ESI) for Chemical Science.
This journal is © The Royal Society of Chemistry 2023

Supplementary Information for:

Sulfur(IV) Reagents for the SuFEx-Based Synthesis of Substituted Sulfamate Esters

Kathleen T. Downey, Jia Y. Mo, Joey Lai, Brodie J. Thomson, and Glenn M. Sammis*

Department of Chemistry, University of British Columbia, 2036 Main Mall, Vancouver, British Columbia V6T 1Z1, Canada

Contents

General methods and instrumentation S3

Representative example of ^{19}F NMR yield determination S4

Sulfur (IV) Fluoride Transfer Reagent Development S5

Solvent and Stability studies of MISF solution S7

General Procedure A: Synthesis of MISF S8

General Procedure B: Synthesis of sulfamidites using MISF S9

Experimental Data S10

References S20

^{1}H, ^{13}C, and ^{19}F NMR Spectra S21

General methods and instrumentation

Materials: All chemicals were used as received from the following commercial sources; Sigma-Aldrich, Bachem, AmBeed, AK Scientific, Alfa Aesar, Oxchem, Advanced ChemTech, and Fluka. All solvents were purchased from commercial sources; Sigma-Aldrich, Fisher Scientific, OmniSolv, and Acros.

All reactions were performed in disposable scintillation 20 mL (28 x 61 mm) glass vials or round-bottom flasks. Screw caps and PTFE/Silicone septa (22 mm x 0.060”) were from Chemglass Life Sciences. Disposable 1 mL syringes (I.D. 4.69 mm), 3 mL Syringes (I.D. 9.65 mm), 5 mL Syringes (I.D. 12.45 mm), 10 mL Syringes (I.D. 15.90 mm), and 30 mL Syringes (I.D. 22.90 mm) were from Norm-Ject and Henk-Ject. Disposable needles 16 G x 1 ½ (1.6 mm x 40 mm) and 21 G x 2 (0.8 mm x 5 mm) were from BD PrecisionGlide™. Hypodermic Needles (22 G x 4”) were from Air-Tite™.

Chromatography: Thin-layer chromatography (TLC) was performed on Merck Silica gel 60 F_{254} TLC aluminium sheets and visualized with 254 nm light and KMnO4 followed by heating.

Infrared spectra (IR) were obtained using a Thermo Nicolet 4700 FT-IR spectrometer or a Perkin Elmer Frontier FT-IR. The spectra are reported in cm^{-1}.

Mass spectrometry: High resolution mass spectra (HRMS) were recorded on a Waters/Micromass LCT spectrometer. Low resolution mass spectra (LRMS) data was obtained using an Agilent 5977A MSD coupled to 7890B GC.

NMR spectra were obtained on a Bruker AV-300 or AV-400 spectrometer. ^{1}H, ^{13}C, and ^{19}F NMR chemical shifts are reported in parts per million (ppm) relative to the residual solvent peak (CDCl3: ^{1}H: δ = 7.26 ppm, ^{13}C: δ = 77.16 ppm). ^{19}F NMR chemical shifts were referenced to $CFCl_3$. NMR yields were determined by ^{19}F NMR using a relaxation time of 40 seconds to complete relaxation of all fluorine nuclei. α, α, α-Trifluorotoluene ($PhCF_3$) was used as an internal standard. Multiplicities are reported as follows: singlet (s), doublet (d), triplet (t), quartet (q), pentet (p), multiplet (m), doublet of doublets (dd), doublet of triplets (dt) and triplet of doublets (td). Assignment of peaks was done based on the chemical shifts, multiplicities, and integrals of the peaks. Coupling constants (J) are reported in Hz.

Representative example for the ^{19}F NMR yield determination

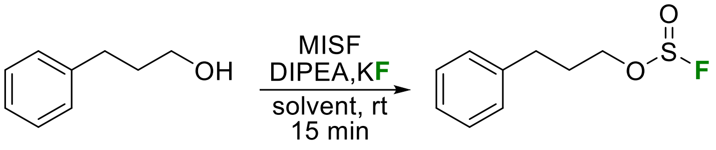

$$\left(\frac{\frac{\text{integration value of product}}{\text{number of nuclei in product}}}{\frac{\text{integration value of internal standard}}{\text{number of nuclei in internal standard}}}\right)\left(\frac{\frac{\text{mass internal standard (g)}}{\text{MW internal standard (g/mol)}}}{\frac{\text{mass substrate (g)}}{\text{MW substrate (g/mol)}}}\right)\text{x100\%} = {}^{19}\text{F NMR yield}$$

$$\left(\frac{\frac{1.00}{\text{1 F nuclei}}}{\frac{0.73}{\text{3 F nuclei}}}\right)\left(\frac{\frac{0.0212\ \text{(g)}}{146.11\ \text{(g/mol)}}}{\frac{0.0816\ \text{(g)}}{136.19\ \text{(g/mol)}}}\right)\text{x}100 = 99\ \%$$

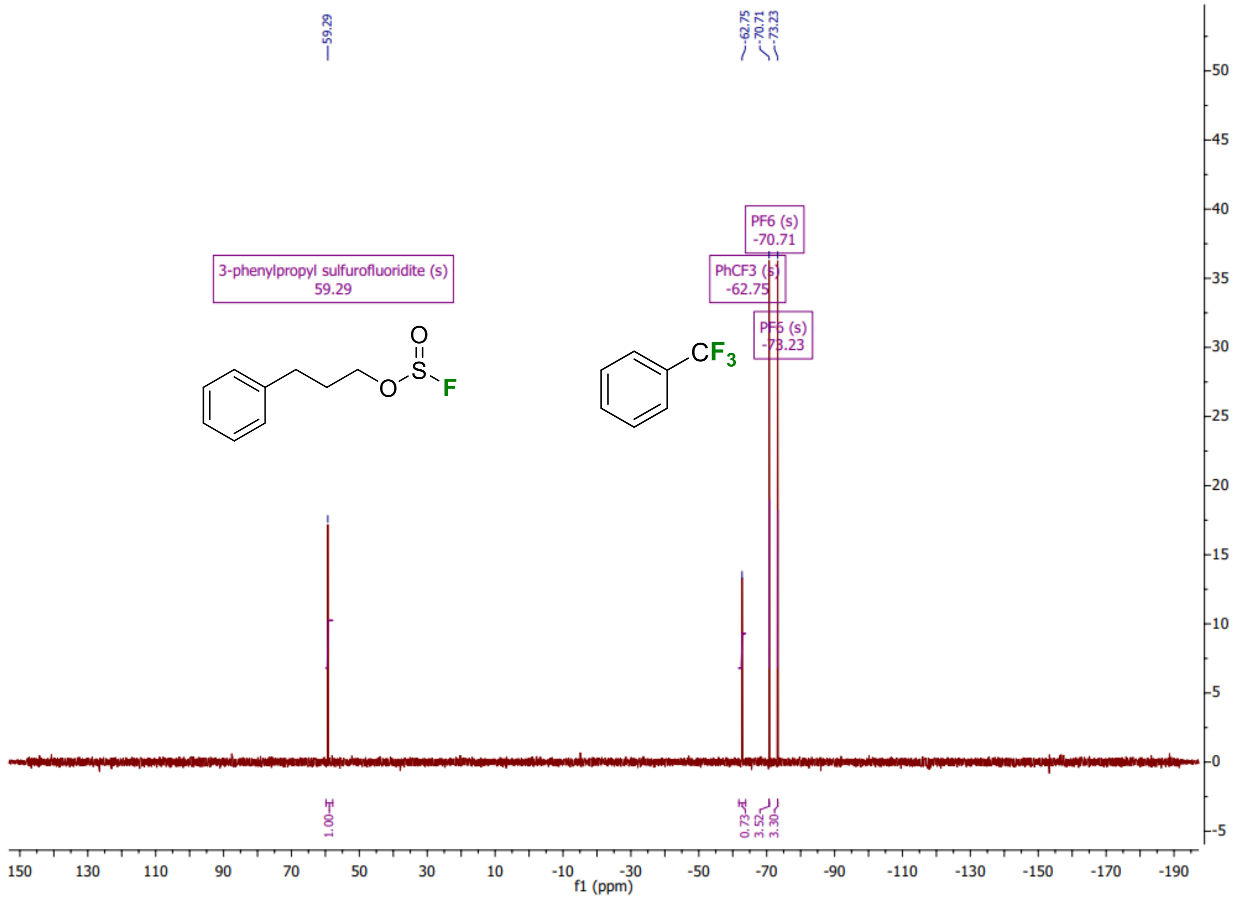

Figure S1. ^{19}F NMR spectrum yield and conversion determination for *p*-toluoyl fluoride **2a**.

Sulfur (IV) Fluoride Transfer Reagent Development

Table S1. Representative structures from reagent structure screen

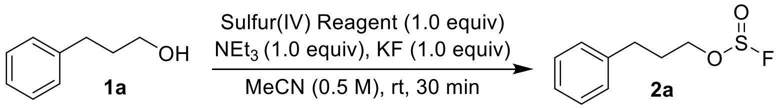

entry	Sulfur(IV) Reagent	yield (%)[a]
1	SOF_2	99%
2	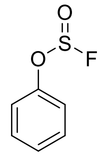	7%
3	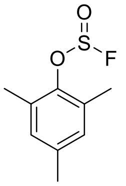	5%
4	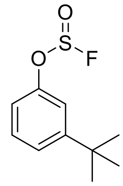	8%
5	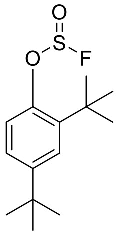	13%
6	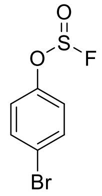	10%
7	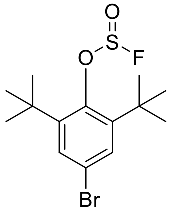	0%
8	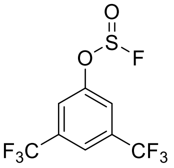	0%
9	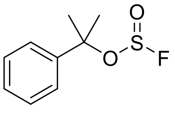	0%
10	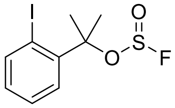	0%
11	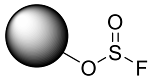	0%[b]
12	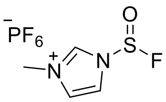	99%
13	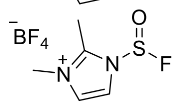	17%
14	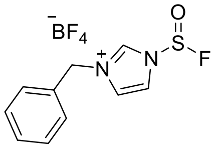	60%
15	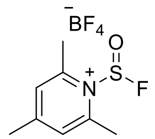	0%
16	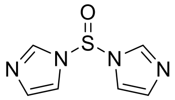	0%
17	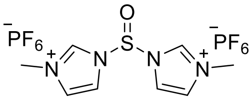	80%
18	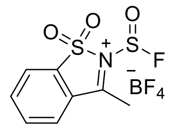	0%
19	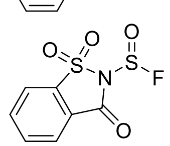	0%
20	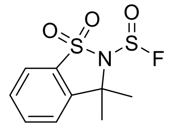	0%

entry	Sulfur(IV) Reagent	yield (%)[a]
21	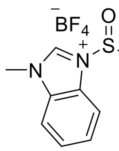	0%
22	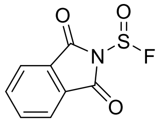	0%
23	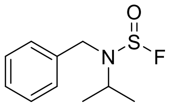	0%
24	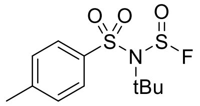	0%
25	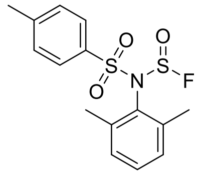	0%
26	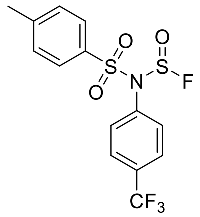	0%
27	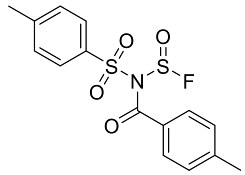	0%
28	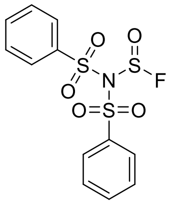	0%
29	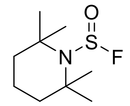	0%
30	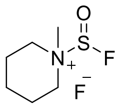	0%
31	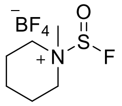	0%
32	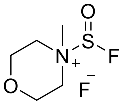	0%
33	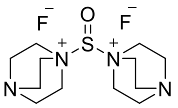	0%
34	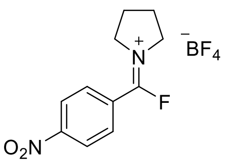	0%
35	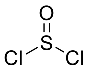	0%
36	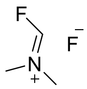	0%

[a]Yields were determined by ^{19}F NMR spectroscopy with trifluorotoluene as an internal standard. [b]The solid support for this reagent was Amberlyst A-26(OH) ion exchange resin.

Optimization study of N-Methylimidazolium Sulfinyl Fluoride Hexafluorophosphate (MISF)

Table S2. Solvent screen for MISF storage

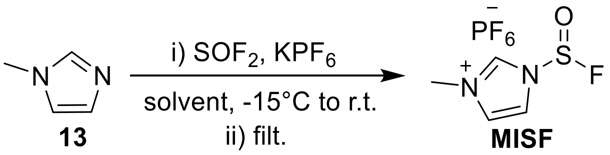

Entry	Solvent	Yield of MISF (%)
1	Et_2O	0
2	DCM	0
3	1,4-dioxane	31
4	MeCN	99
5	THF	98

Reaction conditions: The reactions to form **MISF** were performed on 2 mmol scale of methylimidazole **13** following general procedure A. The yields were determined by ^{19}F NMR spectroscopy using trifluorotoluene as an internal standard.

Table S3. Stability testing of MISF in solution

Entry	Headspace	Time (mo.)	Remaining MISF (%)
1	Argon	1	100
2	Argon	2	100
3	Argon	3	99
4	Air	1	100
5	Air	2	99
6	Air	3	99

MISF was stored in 20 mL glass vials at 0.24 M concentration in MeCN. Yields were determined by ^{19}F NMR spectroscopy using trifluorotoluene as an internal standard.

General Procedure A: Synthesis of N-methylimidazole-1-sulfinic fluoride hexafluorophosphate (MISF)

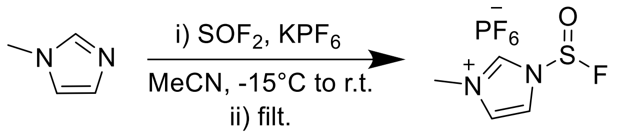

A 100 mL round bottom flask equipped with a magnetic stir-bar and a septum was charged with potassium hexafluorophosphate (2.2099 g, 12.0 mmol) and a solution of SOF_2 in MeCN (59 mL, 0.22 M, 13.0 mmol).[1] The solution was cooled to – 15 °C and methylimidazole (0.96 mL, 12.0 mmol) was added dropwise over 2 minutes. The reaction was warmed to room temperature and stirred for 15 minutes. The mixture was then degassed with Ar for 10 minutes and filtered through Celite to afford the MISF in solution (60 mL, 0.19 M, 11.4 mmol, 95 % yield). The yield was determined by quantitative ^{19}F NMR spectroscopy with trifluorotoluene as the internal standard. ^{1}H NMR (300 MHz, CD_3CN) δ 7.62 (s, 1H), 7.03 (dd, J = 9.0, 3.0 Hz, 2H), 3.69 (s, 3H). $^{19}F\{^{1}H\}$ NMR (282 MHz, CD_3CN) δ 71.59, -72.16, -74.66. $^{13}C\{1H\}$ NMR (75 MHz, CD_3CN) δ 137.66, 127.03, 121.06, 33.27. HRMS-EI+ (*m/z*) calcd for $C_4H_6FN_2OS^+$ ($[M]^+$): 149.0188, found: 149.0185.

General Procedure B: Synthesis of sulfamidites from alcohols and amines using SOF_2 and MISF

Method A: Fluorosulfite intermediate

A 20 mL vial equipped with a magnetic stir-bar and septum-fitted cap was charged with a solution of MISF in MeCN (6 mL), potassium fluoride (0.6 mmol, 1.0 equiv.), alcohol (0.6 mmol, 1 equiv.), and diisopropylethylamine (DIPEA) (90 µL, 0.6 mmol, 1.0 equiv.). The solution was stirred until the reaction was determined to have reached completion by TLC (approximately 15 min). The reaction mixture was then diluted with diethyl ether (5 mL) and filtered through Celite. To the filtrate was then added a mixture of the amine (0.75 mmol, 1.25 equiv.) and DIPEA (110 µL, 0.75 mmol, 1.25 equiv.) in anhydrous MeCN (0.5 mL). The reaction was stirred for 30 minutes.

Method B: Aminosulfinyl fluoride intermediate

A 20 mL vial equipped with a magnetic stir-bar and septum-fitted cap was charged with a solution of MISF in MeCN (6 mL), potassium fluoride (0.6 mmol, 1.0 equiv.), amine (0.6 mmol, 1 equiv.), and diisopropylethylamine (DIPEA) (90 µL, 0.6 mmol, 1.0 equiv.). The solution was stirred until the reaction was determined to have reached completion by TLC (approximately 30 min). The reaction mixture was then diluted with diethyl ether (5 mL) and filtered through Celite. To the filtrate was then added a mixture of the alcohol (0.75 mmol, 1.25 equiv.) and DIPEA (110 µL, 0.75 mmol, 1.25 equiv.) in anhydrous MeCN (0.5 mL). The reaction was stirred for 30 minutes.

Workup:

The reaction mixture was diluted with diethyl ether (15 mL), then washed with water (2 x 15 mL) and brine (15 mL). The organic layer was then dried over Na_2SO_4 and concentrated under reduced pressure.

Conversion of sulfamidites to sulfamate esters

A 20 mL vial equipped with a magnetic stir-bar and a septum-fitted vial cap was charged with $RuCl_3 \bullet H_2O$ (0.05 mmol, 0.10 equiv.), water (0.8 mL), MeCN (2.0 mL), and sodium periodate (1.0 mmol, 1.67 equiv.). The solution was stirred until the solution appeared light brown (approximately 10 minutes). The mixture was cooled to 0 °C and the sulfamidite (0.6 mmol, 1.0 equiv.) diluted in MeCN (2.0 mL) was added. The vessel from which the sulfamidite was then rinsed with MeCN (2 x 1.0 mL) and the rinsings were added to the reaction mixture. The reaction was then stirred until the solution appeared black (approximately 30 minutes). The mixture was then diluted in EtOAc (40 mL) and filtered through Celite. The filtrate was washed with water (2 x 20 mL) and brine (20 mL). The organic layer was dried over Na_2SO_4 and concentrated under reduced pressure.

Experimental Data

3-Phenylpropyl piperidine-1-sulfonate (12a)

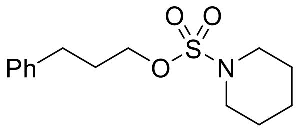

Following the general procedure B using 3-phenylpropanol and piperidine, 3-phenylpropyl piperidine-1-sulfonate (**12a**) was obtained. The product was isolated via flash column chromatography (50% Et_2O/hexane) as a yellow oil.

Yield Method A: 137 mg, 72 % yield, Method B: 99 mg, 52 % yield

^{1}H NMR (300 MHz, Chloroform-*d*) δ 7.36 – 7.25 (m, 2H), 7.27 – 7.14 (m, 3H), 4.16 (t, *J* = 6.4 Hz, 2H), 3.30 – 3.21 (m, 4H), 2.82 – 2.68 (m, 2H), 2.15 – 1.97 (m, 2H), 1.71 – 1.49 (m, 6H);

^{13}C NMR (75 MHz, Chloroform-*d*) δ 140.8, 128.7, 128.6, 126.3, 69.6, 47.7, 31.8, 30.8, 25.1, 23.7;

IR (cm^{-1}) (neat): 2941, 2857, 1364, 1347, 1174;

HRMS-ESI (*m/z*) calcd for $C_{14}H_{21}NO_3SNa^+$ ($[M+Na]^+$): 306.1140, found: 306.1136.

3-Phenylpropyl morpholine-4-sulfonate (12b)

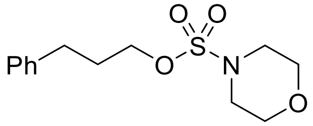

Following the general procedure B using 3-phenylpropanol and morpholine, 3-phenylpropyl morpholine-4-sulfonate (**12b**) was obtained. The product was isolated via flash column chromatography (50% Et_2O/hexane) as a yellow oil. The product contained trace amounts of diethyl ether (<1 wt%) as determined by ^{1}H NMR.

Yield Method A: 138 mg, 73 % yield

^{1}H NMR (300 MHz, Chloroform-*d*) δ 7.37 – 7.25 (m, 2H), 7.27 – 7.14 (m, 3H), 4.20 (t, *J* = 6.4 Hz, 2H), 3.79 – 3.70 (m, 4H), 3.32 – 3.23 (m, 4H), 2.80 – 2.69 (m, 2H), 2.15 – 1.99 (m, 2H);

^{13}C NMR (75 MHz, Chloroform-*d*) δ 140.5, 128.7, 128.6, 126.4, 70.2, 66.1, 46.8, 31.8, 30.7;

IR (cm^{-1}) (neat): 2923, 2862, 1360, 1174, 1114;

HRMS-ESI (*m/z*) calcd for $C_{13}H_{19}NO_4SNa^+$ ($[M+Na]^+$): 308.0933, found: 308.0920.

3-Phenylpropyl 2-methylaziridine-1-sulfonate (12c)

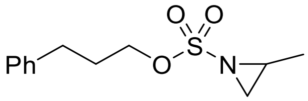

Following the general procedure B using 3-phenylpropanol and 2-methylaziridine, 3-phenylpropyl 2-methylaziridine-1-sulfonate (**12c**) was obtained. The product was isolated via flash column chromatography (30% Et_2O/hexane) as a yellow oil. The product contained trace amounts of diethyl ether (<1 wt%) as determined by ^{1}H NMR.

Yield Method A: 84 mg, 54 % yield

^{1}H NMR (300 MHz, Chloroform-*d*) δ 7.37 – 7.24 (m, 2H), 7.27 – 7.16 (m, 3H), 4.39 (t, *J* = 6.3 Hz, 2H), 2.89 – 2.71 (m, 3H), 2.62 (d, *J* = 7.0 Hz, 1H), 2.18 – 2.02 (m, 3H), 1.33 (d, *J* = 5.6 Hz, 3H);

^{13}C NMR (75 MHz, Chloroform-*d*) δ 140.6, 128.7, 128.6, 126.4, 72.3, 37.0, 35.7, 31.7, 30.8, 16.9;

IR (cm^{-1}) (neat): 2971, 2935, 1353, 1175;

HRMS-ESI (*m/z*) calcd for $C_{12}H_{17}NO_3SNa^+$ ($[M+Na]^+$): 278.0827, found: 278.0816.

3-Phenylpropyl methyl(phenethyl)sulfamate (12d)

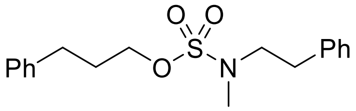

Following the general procedure B using 3-phenylpropanol and N-methyl-2-phenylethylamine, 3-phenylpropyl methyl(phenethyl)sulfamate (**12d**) was obtained. The product was isolated via flash column chromatography (33 % Et_2O/hexane) as a yellow oil.

Yield Method A: 180 mg, 79 % yield, Method B: 153 mg, 71 % yield

^{1}H NMR (300 MHz, Chloroform-*d*) δ 7.36 – 7.22 (m, 4H), 7.25 – 7.12 (m, 6H), 4.05 (t, *J* = 6.3 Hz, 2H), 3.50 – 3.39 (m, 2H), 2.91 (t, *J* = 7.6 Hz, 2H), 2.87 (s, 3H), 2.71 (t, *J* = 7.6 Hz, 2H), 2.08 – 1.93 (m, 2H);

^{13}C NMR (75 MHz, Chloroform-*d*) δ 140.7, 138.3, 129.0, 128.8, 128.7, 128.6, 126.8, 69.6, 52.8, 36.0, 34.4, 31.8, 30.7;

IR (cm^{-1}) (neat): 3028, 2933, 1352, 1167;

HRMS-ESI (*m/z*) calcd for $C_{18}H_{23}NO_3SNa^+$ ($[M+Na]^+$): 356.1296, found: 356.1292.

3-phenylpropyl dibenzylsulfamate (12e)

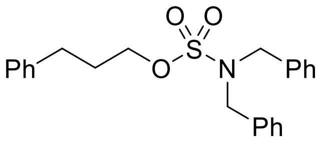

Following the general procedure B using 3-phenylpropanol and dibenzylamine, 3-phenylpropyl dibenzylsulfamate (**12e**) was obtained. The product was isolated via flash column chromatography (20% Et_2O/hexane) as a yellow oil. The product contained trace amounts of diethyl ether (<1 wt%) as determined by ^1H NMR.

Yield Method A: 234 mg, 83 % yield, Method B: 219 mg, 82 % yield

^{1}H NMR (300 MHz, Chloroform-*d*) δ 7.42 – 7.24 (m, 12H), 7.27 – 7.11 (m, 3H), 4.34 (s, 4H), 4.12 (t, *J* = 6.4 Hz, 2H), 2.73 – 2.62 (m, 2H), 2.06 – 1.91 (m, 2H);

^{13}C NMR (75 MHz, Chloroform-*d*) δ 140.8, 135.3, 129.0, 128.8, 128.7, 128.6, 128.2, 126.3, 69.7, 50.9, 31.8, 30.7;

IR (cm^{-1}) (neat): 3030, 2927, 1356, 1167;

HRMS-ESI (*m/z*) calcd for $C_{23}H_{25}NO_3SNa^+$ ($[M+Na]^+$): 418.4553, found: 418.1450.

3-Phenylpropyl diethylsulfamate (12f)

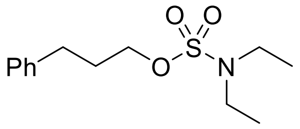

Following the general procedure B using 3-phenylpropanol and diethylamine, 3-phenylpropyl diethylsulfamate (**12f**) was obtained. The product was isolated via flash column chromatography (15% Et_2O/hexane) as a yellow oil.

Yield Method A: 89 mg, 55 % yield

^{1}H NMR (300 MHz, Chloroform-*d*) δ 7.36 – 7.24 (m, 2H), 7.26 – 7.13 (m, 3H), 4.12 (t, *J* = 6.4 Hz, 2H), 3.31 (q, *J* = 7.2 Hz, 4H), 2.78 – 2.67 (m, 2H), 2.11 – 1.96 (m, 2H), 1.20 (t, *J* = 7.2 Hz, 6H);

^{13}C NMR (75 MHz, Chloroform-*d*) δ 140.8, 128.7, 128.6, 126.3, 69.1, 42.8, 31.9, 30.8, 13.5;

IR (cm^{-1}) (neat): 2977, 1357, 1162;

HRMS-ESI (*m/z*) calcd for $C_{13}H_{21}NO_3SNa^+$ ($[M+Na]^+$): 294.1140, found: 294.1136.

3-Phenylpropyl methoxy(methyl)sulfamate (12g)

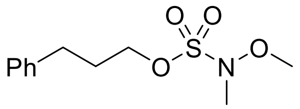

Following the general procedure B using 3-phenylpropanol and *N,O*-dimethylhydroxylamine, 3-phenylpropyl methoxy(methyl)sulfamate (**12g**) was obtained. The product was isolated via flash column chromatography (50% Et_2O/hexane) as a deep red oil. The product contained trace amounts of diethyl ether (<1 wt%) and *N,O*-dimethylhydroxylamine (<1 wt%) as determined by ^{1}H NMR.

Yield Method A: 107 mg, 63 % yield

^{1}H NMR (300 MHz, Chloroform-*d*) δ 7.36 – 7.25 (m, 2H), 7.27 – 7.16 (m, 3H), 4.43 (t, *J* = 6.3 Hz, 2H), 3.76 (s, 3H), 3.06 (s, 3H), 2.82 – 2.69 (m, 2H), 2.17 – 2.02 (m, 2H);

^{13}C NMR (75 MHz, Chloroform-*d*) δ 140.6, 128.7, 128.6, 126.4, 74.1, 63.4, 39.8, 31.7, 31.1;

IR (cm^{-1}) (neat): 2941, 1375, 1190;

HRMS-EI (*m/z*) calcd for $C_{11}H_{17}NO_4S^+$ ($[M]^+$): 259.0878, found: 259.0883.

3-Phenylpropyl (4-methoxybenzyl)(propyl)sulfamate (12h)

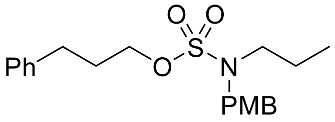

Following the general procedure B using 3-phenylpropanol and N-(4-methoxybenzyl)propan-1-amine, 3-phenylpropyl (4-methoxybenzyl)(propyl)sulfamate (**12h**) was obtained. The product was isolated via flash column chromatography (20% Et_2O/hexane) as an orange oil.

Yield Method A: 139 mg, 60 % yield, Method B: 126 mg, 57 % yield

^{1}H NMR (300 MHz, Chloroform-*d*) δ 7.36 – 7.20 (m, 4H), 7.24 – 7.13 (m, 2H), 6.93 – 6.82 (m, 2H), 4.35 (s, 2H), 4.13 (t, *J* = 6.4 Hz, 2H), 3.80 (s, 3H), 3.15 – 3.04 (m, 2H), 2.77 – 2.66 (m, 2H), 2.10 – 1.95 (m, 2H), 1.64 – 1.46 (m, 2H), 0.83 (t, *J* = 7.4 Hz, 3H);

^{13}C NMR (75 MHz, Chloroform-*d*) δ 159.5, 140.8, 130.0, 128.7, 128.6, 127.9, 126.3, 114.1, 69.4, 55.4, 51.4, 49.6, 31.9, 30.8, 20.8, 11.2;

IR (cm^{-1}) (neat): 2963, 1935, 1512, 1353, 1246, 1165;

HRMS-ESI (*m/z*) calcd for $C_{20}H_{27}NO_4SNa^+$ ($[M+Na]^+$): 400.1559, found: 400.1421.

3-phenylpropyl propylsulfamate (14)

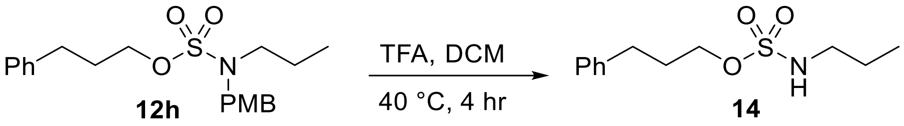

To a solution of 3-phenylpropyl (4-methoxybenzyl)(propyl)sulfamate (**12h**) (0.3 mmol) in 1.5 mL DCM was charged 1.5 mL trifluoracetic acid. The resulted reaction mixture was stirred at 40 ℃ for four hours. The solution was diluted with EtOAc (20 mL), then was washed with sat. $NaHCO_3$ (50 mL). The organic layer was washed with H_2O (3 x 50 mL), brine (50 mL), dried over Na_2SO_4 and concentrated under reduced pressure. The product (**14**) was isolated via flash column chromatography (30% Et_2O/petroleum ether) as a yellow oil (77 mg, 100% yield). The product contained trace amounts of diethyl ether (<1 wt%) as determined by ^{1}H NMR.

^{1}H NMR (300 MHz, Chloroform-*d*) δ 7.36 – 7.25 (m, 2H), 7.27 – 7.15 (m, 3H), 4.32 (s, 1H), 4.14 (t, *J* = 6.3 Hz, 2H), 3.16 – 3.03 (m, 2H), 2.80 – 2.69 (m, 2H), 2.14 – 1.98 (m, 2H), 1.66 – 1.50 (m, 2H), 0.95 (t, *J* = 7.4 Hz, 3H);

^{13}C NMR (75 MHz, Chloroform-*d*) δ 140.7, 128.7, 128.6, 126.4, 69.8, 45.8, 31.8, 30.6, 23.0, 11.2;

IR (cm^{-1}) (neat): 3306, 2966, 2878, 1344, 1170;

HRMS-ESI (*m/z*) calcd for $C_{12}H_{19}NO_3SNa^+$ ($[M+Na]^+$): 280.0983, found: 280.0979.

3-phenylpropyl (S)-2-carbamoylpyrrolidine-1-sulfonate (12i)

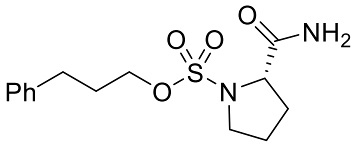

Following the general procedure B using 3-phenylpropanol and (S)-pyrrolidine-2-carboxamide, 3-phenylpropyl (S)-2-carbamoylpyrrolidine-1-sulfonate (**12i**) was obtained. The product was isolated via flash column chromatography (60% EtOAc in DCM) as a white solid.

Yield Method A: 111 mg, 60 % yield

^{1}H NMR (300 MHz, Chloroform-*d*) δ 7.37 – 7.29 (m, 2H), 7.27 – 7.17 (m, 3H), 6.58 (s, 1H), 5.68 (s, 1H), 4.37 – 4.19 (m, 3H), 3.58 – 3.44 (m, 2H), 2.75 (t, *J* = 7.5 Hz, 2H), 2.41 – 2.31 (m, 1H), 2.21 – 2.05 (m, 3H), 2.02 – 1.85 (m, 2H);

^{13}C NMR (75 MHz, Chloroform-*d*) δ 173.5, 140.4, 128.7, 128.5, 126.5, 70.5, 62.9, 50.7, 31.8, 30.7, 30.4, 25.0;

IR (cm^{-1}) (neat): 3437, 3177, 2951, 1627, 1355, 1166;

HRMS-ESI (*m/z*) calcd for $C_{14}H_{20}N_2O_4SNa^+$ ($[M+Na]^+$): 355.1042, found: 355.1036.

3-phenylpropyl 4-(6-(trifluoromethyl)pyridin-2-yl)piperazine-1-sulfonate (12j)

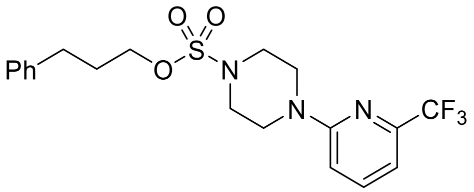

Following the general procedure B using 3-phenylpropanol and 1-(6-(trifluoromethyl)pyridin-2-yl)piperazine, 3-phenylpropyl 4-(6-(trifluoromethyl)pyridin-2-yl)piperazine-1-sulfonate (**12j**) was obtained. The product was isolated via flash column chromatography (50 % Et_2O/hexane) as a white solid.

Yield Method A: 203 mg, 79 % yield

^{1}H NMR (300 MHz, Chloroform-*d*) δ 8.42 (s, 1H), 7.68 (dd, *J* = 9.0, 2.5 Hz, 1H), 7.32 – 7.26 (m, 2H), 7.23 – 7.14 (m, 3H), 6.67 (d, *J* = 9.0 Hz, 1H), 4.21 (t, *J* = 6.4 Hz, 2H), 3.80 – 3.70 (m, 4H), 3.43 – 3.33 (m, 4H), 2.73 (t, *J* = 7.5 Hz, 2H), 2.14 – 1.99 (m, 2H).

^{13}C NMR (75 MHz, Chloroform-*d*) δ 159.8, 145.7, 140.4, 134.9, 128.6, 128.42, 126.3, 105.9, 70.1, 46.3, 44.1, 31.6, 30.6;

^{19}F NMR (282 MHz, Chloroform-*d*) δ -61.6 (s)

IR (cm^{-1}) (neat): 2921, 2852, 1614, 1331, 1248, 1168;

HRMS-EI (*m/z*) calcd for $C_{19}H_{22}F_3N_3O_3S^+$ ($[M]^+$): 429.1334 found: 429.1338

4-Fluorobenzyl dibenzylsulfamate (12k)

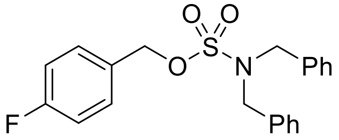

Following the general procedure B using (4-fluorophenyl)methanol and dibenzylamine, 4-Fluorobenzyl dibenzylsulfamate (**12k**) was obtained. The product was isolated via flash column chromatography (33 % Et_2O/hexane) as a white solid.

Yield Method A: 147 mg, 63 % yield, Method B: 121 mg, 52 % yield

^{1}H NMR (300 MHz, Chloroform-*d*) δ 7.37 – 7.17 (m, 12H), 7.05 (t, *J* = 8.6 Hz, 2H), 5.07 (s, 2H), 4.34 (s, 4H);

^{13}C NMR (75 MHz, Chloroform-*d*) δ 163.2 (d, *J* = 248.2 Hz), 135.2, 130.7 (d, *J* = 8.4 Hz), 128.9 (d, *J* = 18.5 Hz), 128.3, 115.8 (d, *J* = 21.7 Hz), 71.2, 50.9;

^{19}F NMR (282 MHz, Chloroform-*d*) δ -112.7 (tt, *J* = 9.4, 5.3 Hz);

IR (cm^{-1}) (neat): 3029, 1368, 1167;

HRMS-EI (*m/z*) calcd for $C_{21}H_{20}FNO_3S^+$ ($[M]^+$): 385.1148, found: 385.1159.

4-nitrophenethyl dibenzylsulfamate (12l)

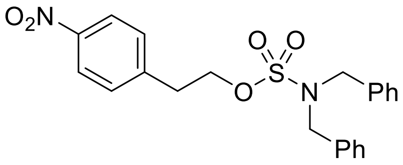

Following the general procedure B using 2-(4-nitrophenyl)ethan-1-ol and dibenzylamine, 4-nitrophenethyl dibenzylsulfamate (**12l**) was obtained. The product was isolated via flash column chromatography (50% Et_2O/hexane) as a yellow solid.

Yield Method A: 200 mg, 79 % yield, Method B: 184 mg, 70 % yield

^{1}H NMR (300 MHz, Chloroform-*d*) δ 8.19 – 8.09 (m, 2H), 7.37 – 7.26 (m, 8H), 7.29 – 7.16 (m, 4H), 4.33 (t, *J* = 6.5 Hz, 2H), 4.26 (s, 4H), 3.07 (t, *J* = 6.5 Hz, 2H);

^{13}C NMR (75 MHz, Chloroform-*d*) δ 147.2, 144.6, 135.0, 130.0, 128.9, 128.8, 128.3, 123.9, 69.3, 50.8, 35.3;

IR (cm^{-1}) (neat): 3032, 2941, 1510 ,1371, 1347, 1168;

HRMS-ESI (*m/z*) calcd for $C_{22}H_{22}N_2O_5SNa^+$ ($[M+Na]^+$): 449.1147, found: 449.1136.

4-(benzyloxy)phenethyl dibenzylsulfamate (12m)

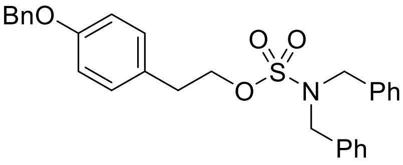

Following the general procedure B using 4-phenylbutan-2-ol and dibenzylamine, 4-(benzyloxy)phenethyl dibenzylsulfamate (**12m**) was obtained. The product was isolated via flash column chromatography (20 % Et_2O/hexane) as an orange oil.

Yield Method A: 187 mg, 63 % yield, Method B: 193 mg, 65 % yield

^{1}H NMR (300 MHz, Chloroform-*d*) δ 7.48 – 7.26 (m, 11H), 7.27 – 7.17 (m, 4H), 7.11 – 7.01 (m, 2H), 6.95 – 6.85 (m, 2H), 5.04 (s, 2H), 4.25 (s, 6H), 2.91 (t, *J* = 7.0 Hz, 2H);

^{13}**C NMR** (75 MHz, Chloroform-*d*) δ 157.9, 137.1, 135.3, 130.2, 129.0, 128.8, 128.2, 128.1, 127.6, 115.1, 70.8, 70.2, 50.8, 34.6;

IR (cm^{-1}) (neat): 3032, 2930, 1511, 1357, 1167;

HRMS-ESI (*m/z*) calcd for $C_{29}H_{29}NO_4SNa^+$ ($[M+Na]^+$): 510.1715, found: 510.1709.

4-phenylbutan-2-yl dibenzylsulfamate (12n)

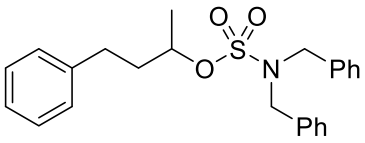

Following the general procedure B using 2-(4-(benzyloxy)phenyl)ethan-1-ol and dibenzylamine, 4-phenylbutan-2-yl dibenzylsulfamate (**12n**) was obtained. The product was isolated via flash column chromatography (15 % Et_2O/hexane) as an orange oil.

Yield Method A: 192 mg, 78 % yield, Method B: 128 mg, 52 % yield

^{1}**H NMR** (300 MHz, Chloroform-*d*) δ 7.41 – 7.23 (m, 12H), 7.26 – 7.13 (m, 3H), 4.76 (dq, *J* = 13.0, 6.3 Hz, 1H), 4.33 (d, *J* = 2.3 Hz, 4H), 2.80 – 2.57 (m, 2H), 2.10 – 1.80 (m, 2H), 1.42 (d, *J* = 6.3 Hz, 3H);

^{13}**C NMR** (75 MHz, Chloroform-*d*) δ 141.3, 135.4, 129.0, 128.7, 128.6, 128.5, 128.1, 126.2, 79.7, 50.8, 38.5, 31.4, 20.9;

IR (cm^{-1}) (neat): 3030, 2932, 1344, 1168;

HRMS-ESI (*m/z*) calcd for $C_{24}H_{27}NO_3SNa^+$ ($[M+Na]^+$): 432.1609, found: 432.1598.

(S)-1-(benzyloxy)propan-2-yl dibenzylsulfamate (12o)

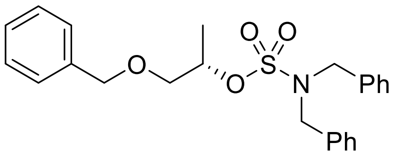

Following the general procedure B using (S)-1-(benzyloxy)propan-2-ol and dibenzylamine, (S)-1-(benzyloxy)propan-2-yl dibenzylsulfamate (**12o**) was obtained. The product was isolated via flash column chromatography (25% Et_2O/Hexanes) as a clear oil.

Yield Method A: 175 mg, 70 % yield

^{1}**H NMR** (300 MHz, Chloroform-*d*) δ 7.39 – 7.20 (m, 15H), 4.85 (dq, *J* = 12.1, 6.3 Hz, 1H), 4.60 – 4.45 (m, 2H), 4.31 (s, 4H), 3.64 – 3.48 (m, 2H), 1.41 (d, *J* = 6.5 Hz, 3H);

^{13}**C NMR** (75 MHz, Chloroform-*d*) δ 138.0, 135.4, 129.0, 128.6, 128.6, 128.0, 127.9, 127.8, 78.1, 73.5, 72.6, 51.0, 17.8;

IR (cm^{-1}) (neat): 3032, 2928, 2866, 1355, 1170;

HRMS-ESI (*m/z*) calcd for $C_{24}H_{27}NO_4SNa^+$ ($[M+Na]^+$): 448.1559, found: 448.1550.

(1R,2S,5R)-2-isopropyl-5-methylcyclohexyl dibenzylsulfamate (12p)

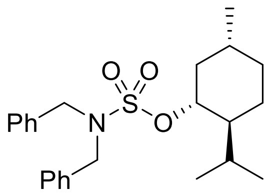

Following the general procedure B using (1R,2S,5R)-2-isopropyl-5-methylcyclohexan-1-ol and dibenzylamine, (1R,2S,5R)-2-isopropyl-5-methylcyclohexyl dibenzylsulfamate (**12p**) was obtained. The product was isolated via flash column chromatography (20% Et_2O/hexane) as a colourless oil.

Yield Method A: 162 mg, 67 % yield

^{1}H NMR (300 MHz, Chloroform-*d*) δ 7.39 – 7.24 (m, 10H), 4.45 (dd, *J* = 10.8, 4.5 Hz, 1H), 4.37 (d, *J* = 15.4 Hz, 2H), 4.25 (d, *J* = 15.3 Hz, 2H), 2.42 – 2.29 (m, 1H), 2.17 – 1.96 (m, 1H), 1.77 – 1.61 (m, 2H), 1.53 – 0.94 (m, 5H), 0.92 (d, *J* = 6.5 Hz, 3H), 0.89 (d, *J* = 7.1 Hz, 3H), 0.82 (d, *J* = 6.9 Hz, 3H).

^{13}C NMR (75 MHz, Chloroform-*d*) δ 135.5, 129.0, 128.6, 128.1, 83.5, 50.8, 48.0, 41.9, 34.0, 31.8, 25.7, 23.2, 22.1, 21.1, 15.8;

IR (cm^{-1}) (neat): 2955, 2870, 1357, 1169;

HRMS-ESI (*m/z*) calcd for $C_{24}H_{33}NO_3SNa^+$ ($[M+Na]^+$): 438.2079, found: 438.2074.

pyridin-4-ylmethyl dibenzylsulfamate (12q)

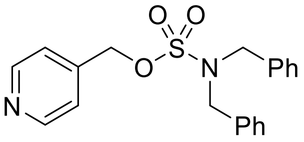

Following the general procedure B using pyridin-4-ylmethanol and dibenzylamine, pyridin-4-ylmethyl dibenzylsulfamate (**12q**) was obtained. The product was isolated via flash column chromatography (40% Et_2O/DCM) as a green solid.

Yield Method A: 90 mg, 40 % yield

^{1}H NMR (300 MHz, Chloroform-*d*) δ 8.62 (d, *J* = 6.2 Hz, 2H), 7.41 – 7.23 (m, 10H), 7.19 (d, *J* = 6.1 Hz, 2H), 5.10 (s, 2H), 4.38 (s, 4H);

^{13}C NMR (75 MHz, Chloroform-*d*) δ 149.4, 135.0, 129.0, 128.9, 128.5, 122.2, 69.1, 51.1;

IR (cm^{-1}) (neat): 3068, 3029, 1371, 1168;

HRMS-ESI (*m/z*) calcd for $C_{20}H_{21}NO_3S^+$ ($[M+H]^+$): 369.1293, found: 369.1265.

((3aR,5R,5aS,8aS,8bR)-2,2,7,7-tetramethyltetrahydro-5H-bis([1,3]dioxolo)[4,5-b:4',5'-d]pyran-5-yl)methyl dibenzylsulfamate (12r)

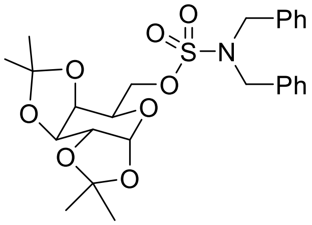

Following the general procedure B using ((3aR,5R,5aS,8aS,8bR)-2,2,7,7-tetramethyltetrahydro-5H-bis([1,3]dioxolo)[4,5-b:4',5'-d]pyran-5-yl)methanol and dibenzylamine, ((3aR,5R,5aS,8aS,8bR)-2,2,7,7-tetramethyltetrahydro-5H-bis([1,3]dioxolo)[4,5-b:4',5'-d]pyran-5-yl)methyl dibenzylsulfamate (**12r**) was obtained. The product was isolated via flash column chromatography (33% Et_2O/hexane) as a yellow oil. The product contained trace amounts of diethyl ether (<1 wt%) as determined by ^1H NMR.

Yield Method A: 161 mg, 53 % yield

^{1}H NMR (300 MHz, Chloroform-*d*) δ 7.40 – 7.24 (m, 10H), 5.55 (d, *J* = 5.0 Hz, 1H), 4.63 (dd, *J* = 7.7, 2.5 Hz, 1H), 4.43 – 4.27 (m, 6H), 4.32 – 4.11 (m, 3H), 1.53 (s, 3H), 1.45 (s, 3H), 1.34 (s, 6H);

^{13}C NMR (75 MHz, Chloroform-*d*) δ 135.2, 129.0, 128.7, 128.1, 109.8, 109.1, 96.4, 70.9, 70.8, 70.5, 68.9, 66.2, 50.8, 26.1, 26.1, 25.1, 24.6;

IR (cm^{-1}) (neat): 2988, 2935, 1365, 1210, 1169, 1067;

HRMS-ESI (*m/z*) calcd for $C_{26}H_{34}NO_8S^+$ ($[M+H]^+$): 520.2005, found: 520.2006.

Unsuccessful substrates

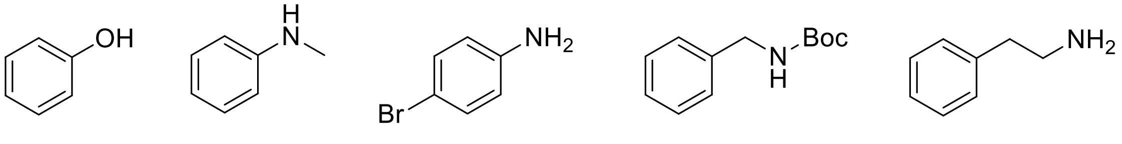

1s **10s** **10t** **10u** **10v**

Following the general procedure A using phenol (**1s**) and piperidine (**10a**), the fluorosulfite was obtained but not the corresponding sulfamidite. Following the general procedure A using 3-phenylpropanol and *N*-methylaniline (**10s**), the desired product was not obtained. Following a modified general procedure A (substituting MISF with SOF_2) using 3-phenylpropanol and either *N*-methylaniline (**10s**), 4-bromoaniline (**10t**), *tert*-butyl benzylcarbamate (**10u**), or 2-phenylethanamine (**10v**), the desired product was not obtained.

References

1. Thionyl fluoride solution was prepared as described in C. Lee, B. J. Thomson, G. M. Sammis, *Chem. Sci.* **2022**, *13*, 188–194.

MISF ^{1}H NMR (300 MHz, Acetonitrile-*d3*)

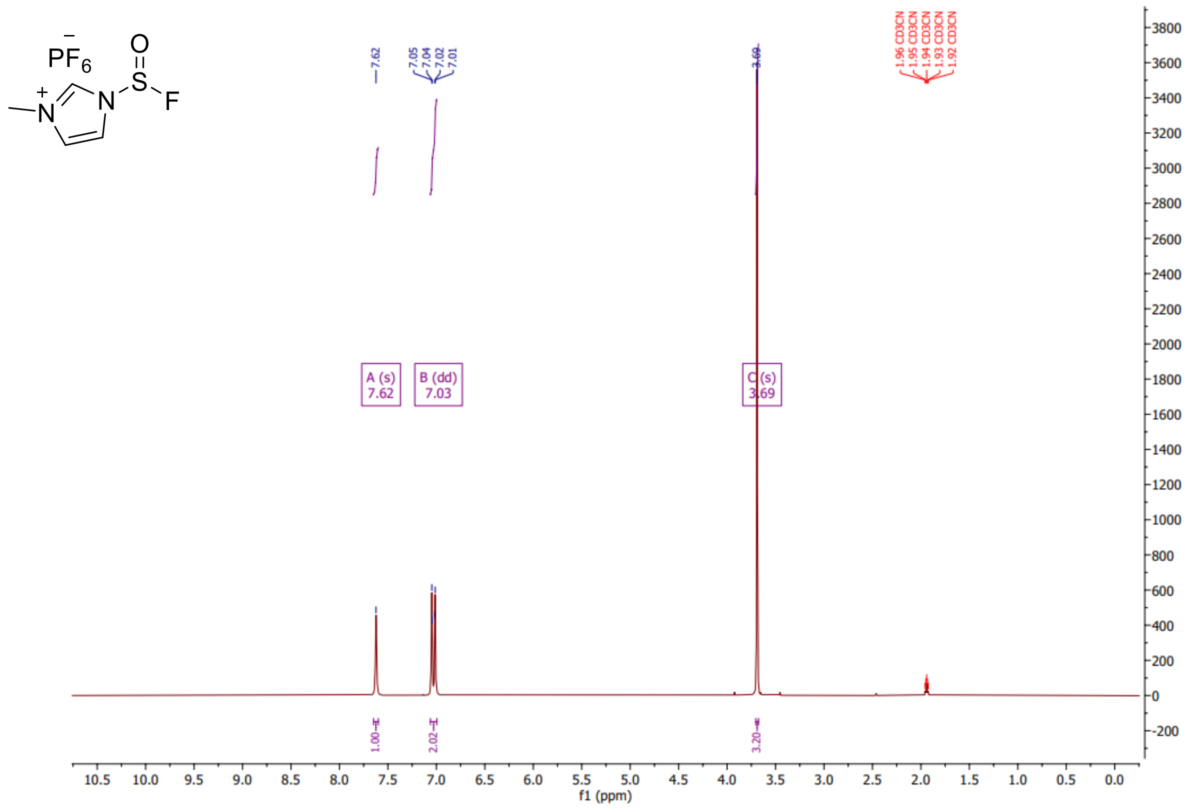

MISF ^{13}C{^{1}H} NMR (75 MHz, Acetonitrile-*d3*)

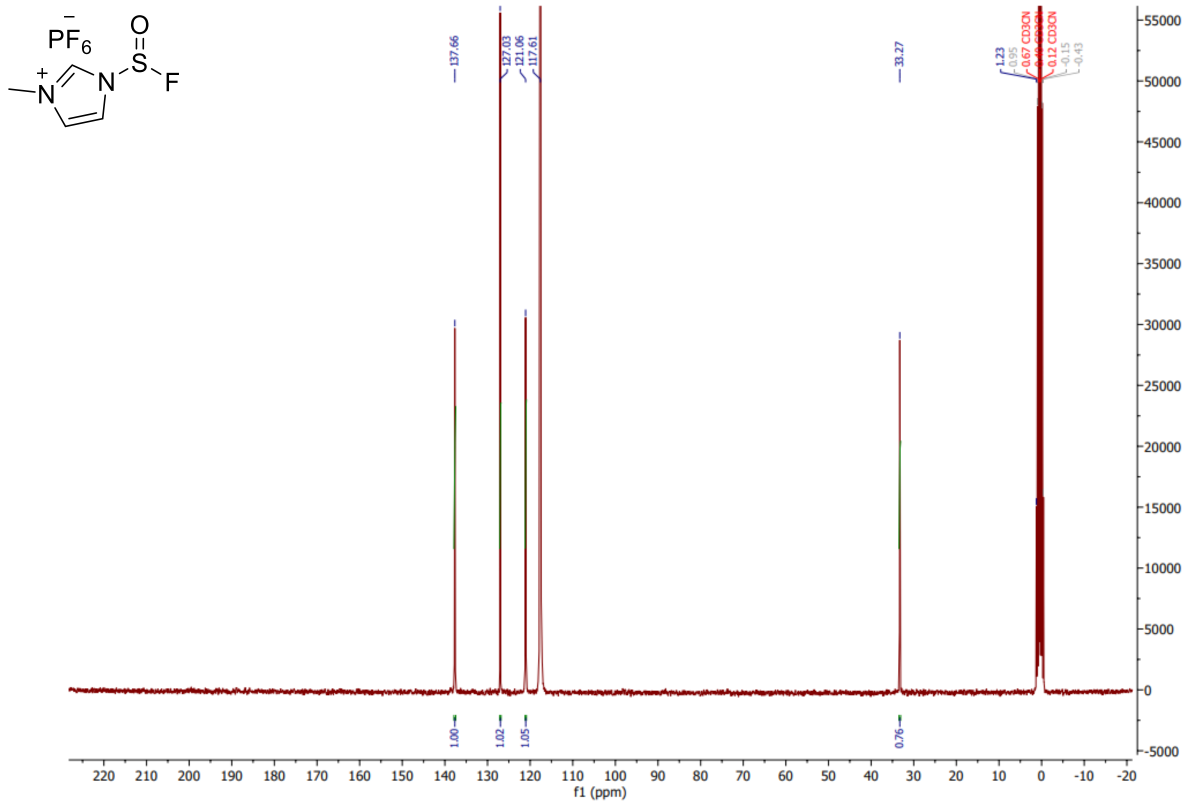

MISF $^{19}F\{^{1}H\}$ NMR (282 MHz, Acetonitrile-*d3*)

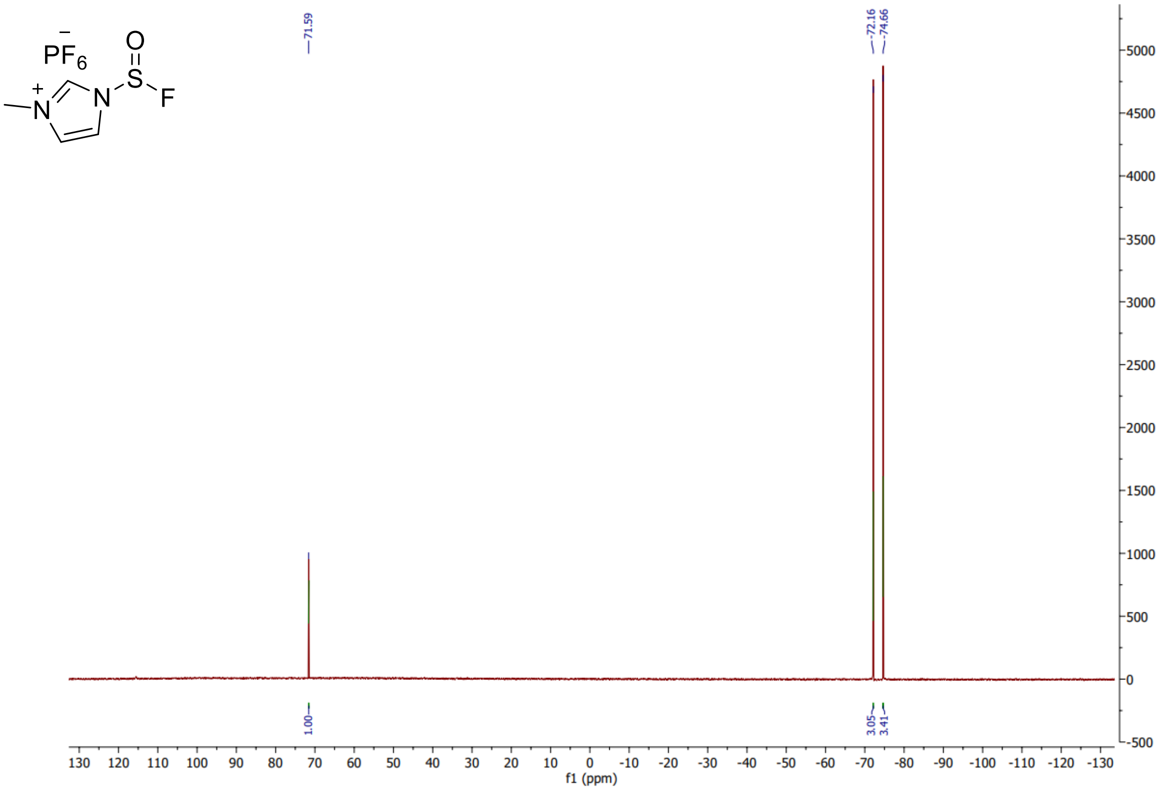

12a ^{1}H NMR (300 MHz, Chloroform-*d*)

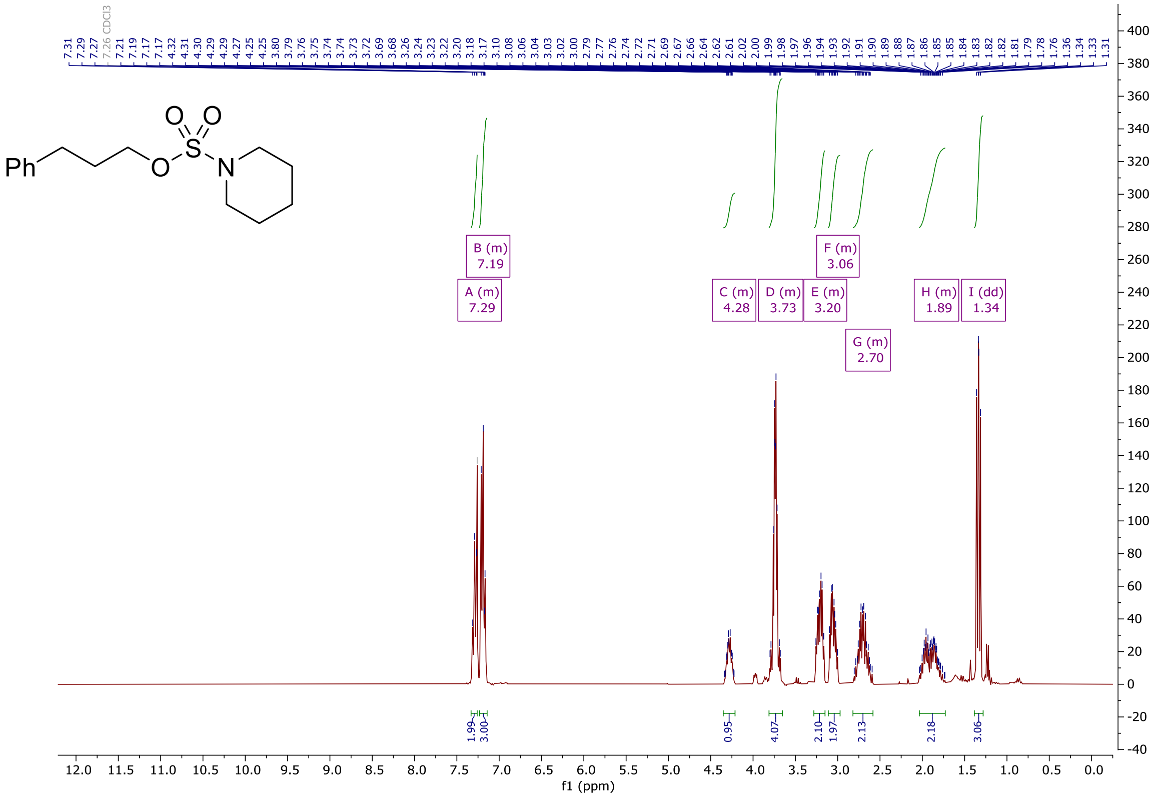

12a ^{13}C{^{1}H} NMR (75 MHz, Chloroform-*d*)

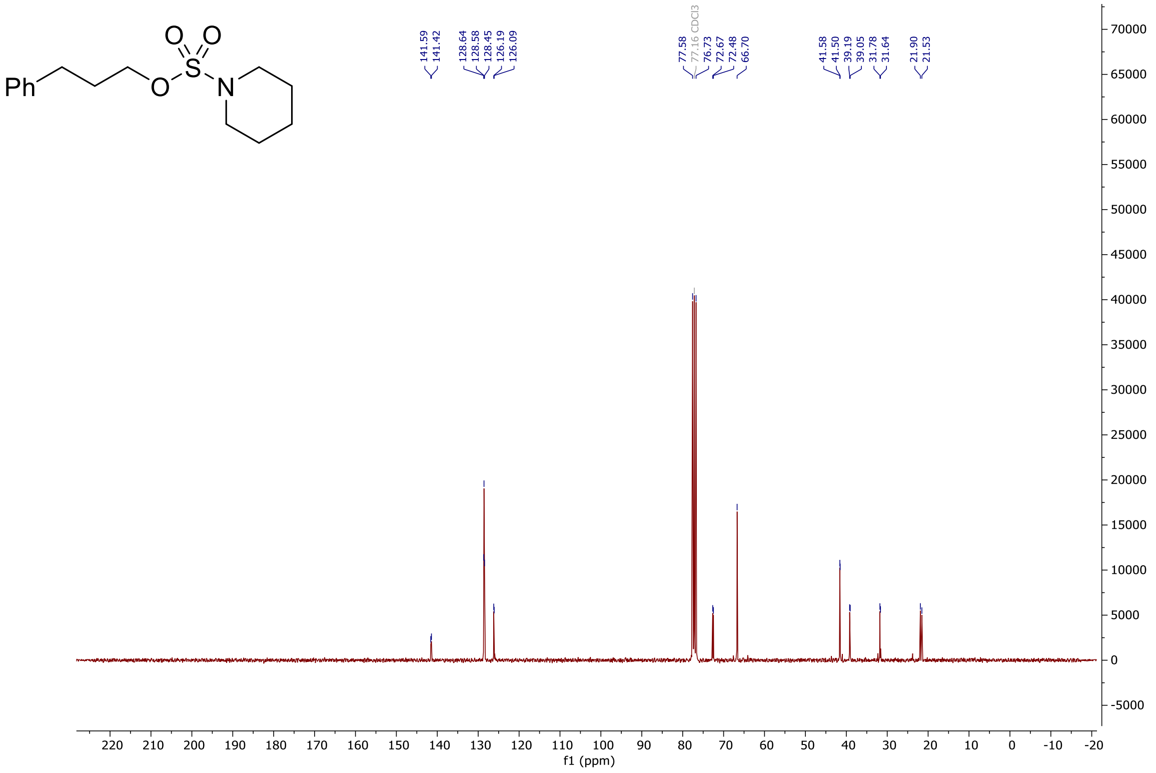

12b ^{1}H NMR (300 MHz, Chloroform-*d*)

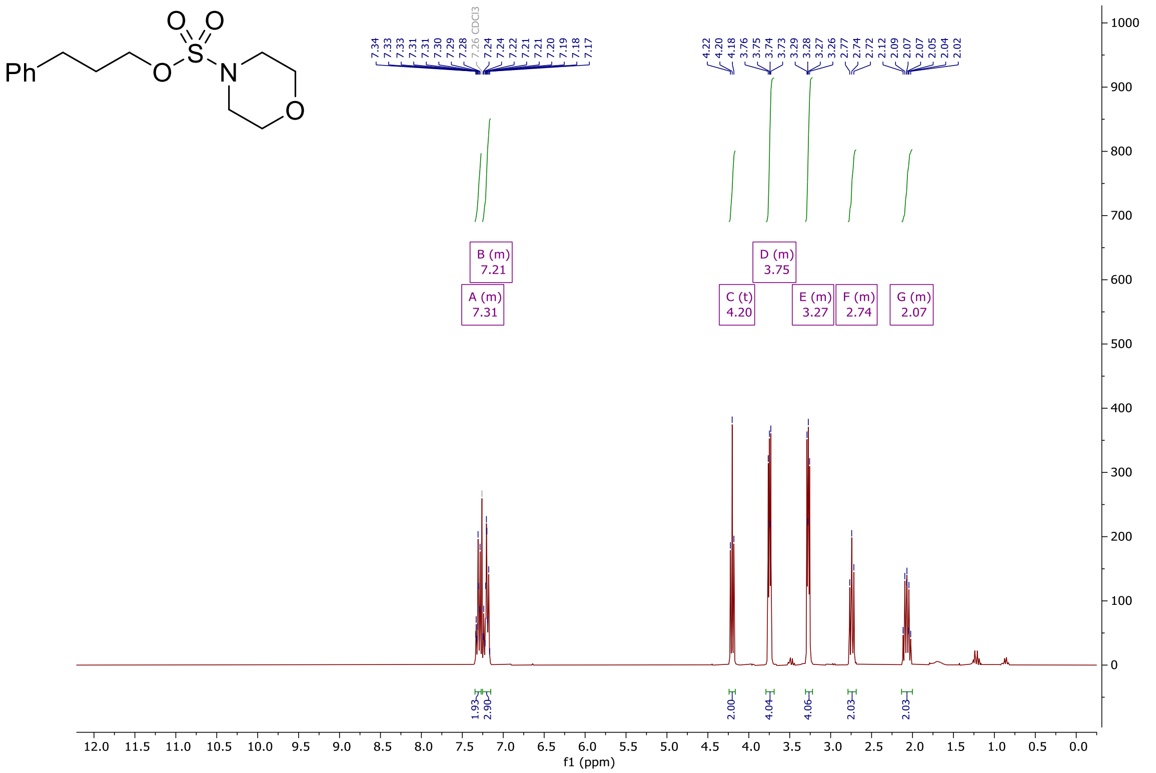

12b $^{13}C\{^{1}H\}$ NMR (75 MHz, Chloroform-*d*)

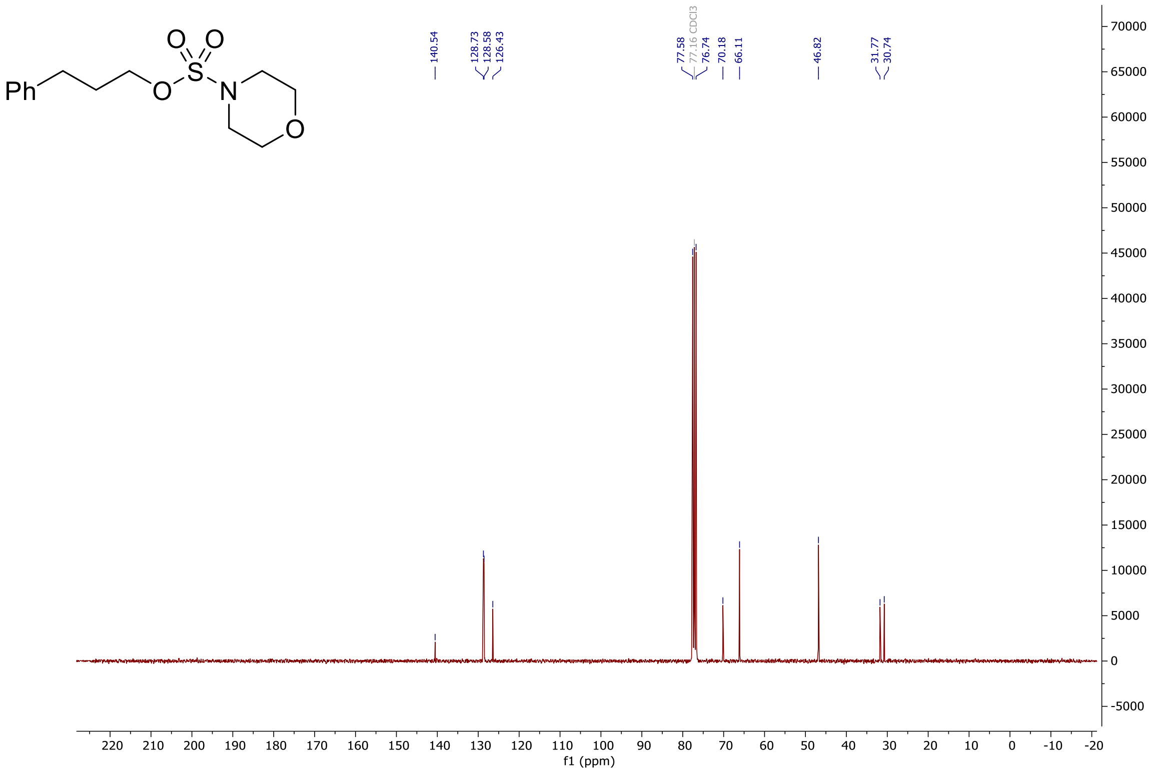

12c ^{1}H NMR (300 MHz, Chloroform-*d*)

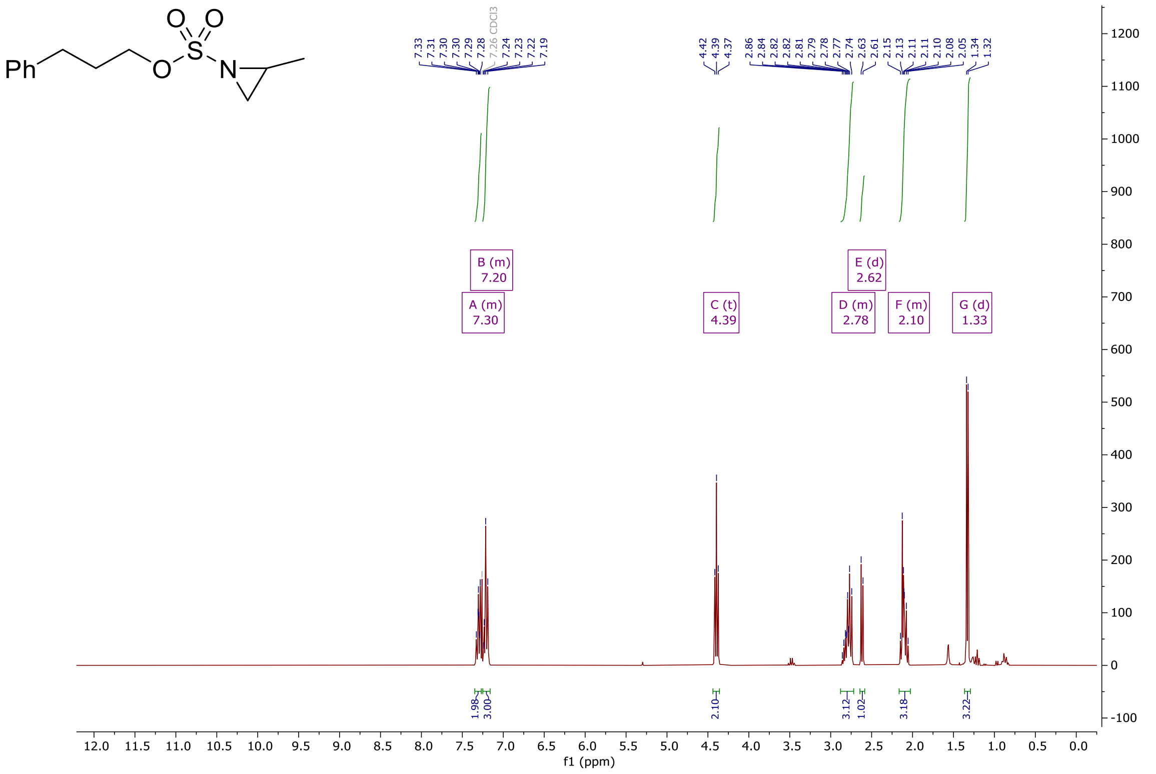

12c ^{13}C{^{1}H} NMR (75 MHz, Chloroform-*d*)

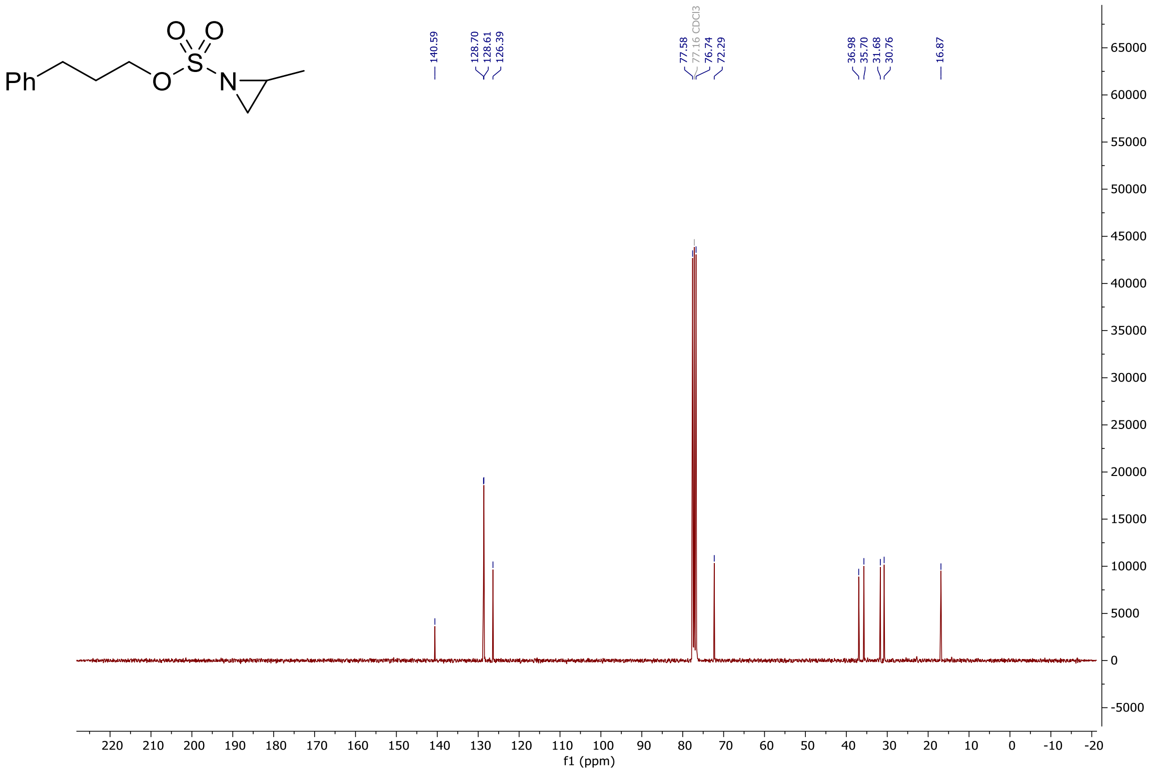

12d ^{1}H NMR (300 MHz, Chloroform-*d*)

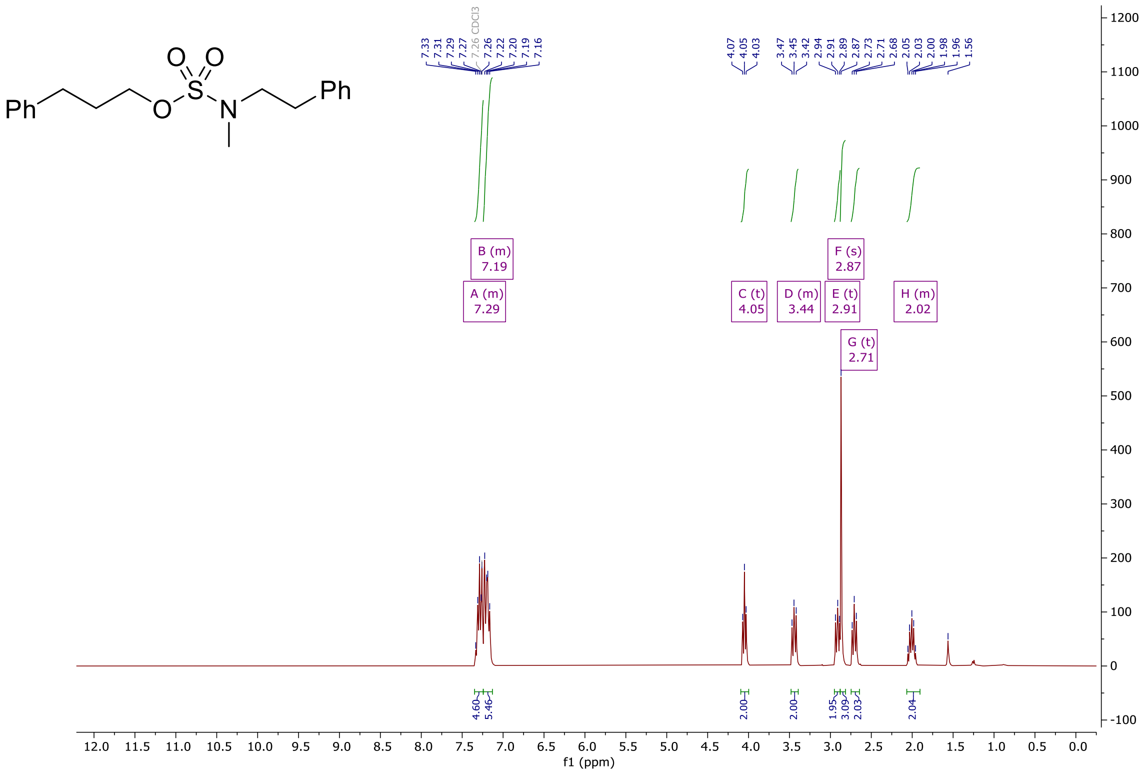

12d ^{13}C{^{1}H} NMR (75 MHz, Chloroform-*d*)

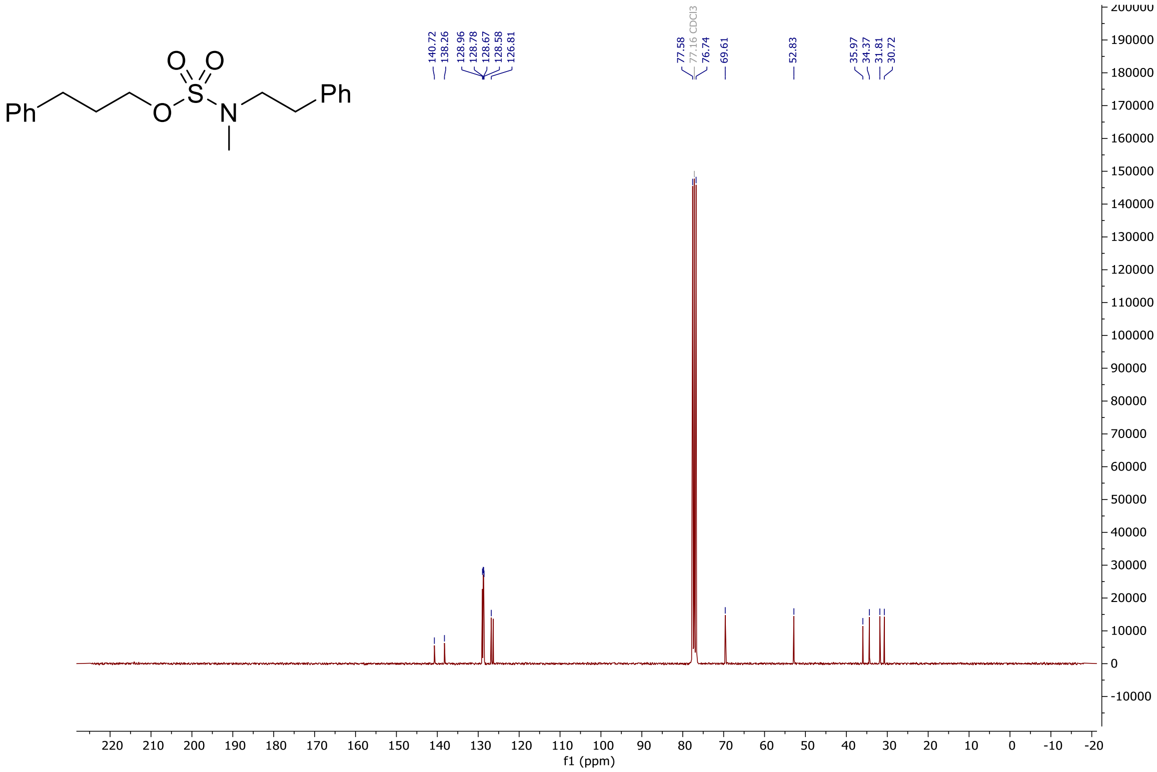

12e ^{1}H NMR (300 MHz, Chloroform-*d*)

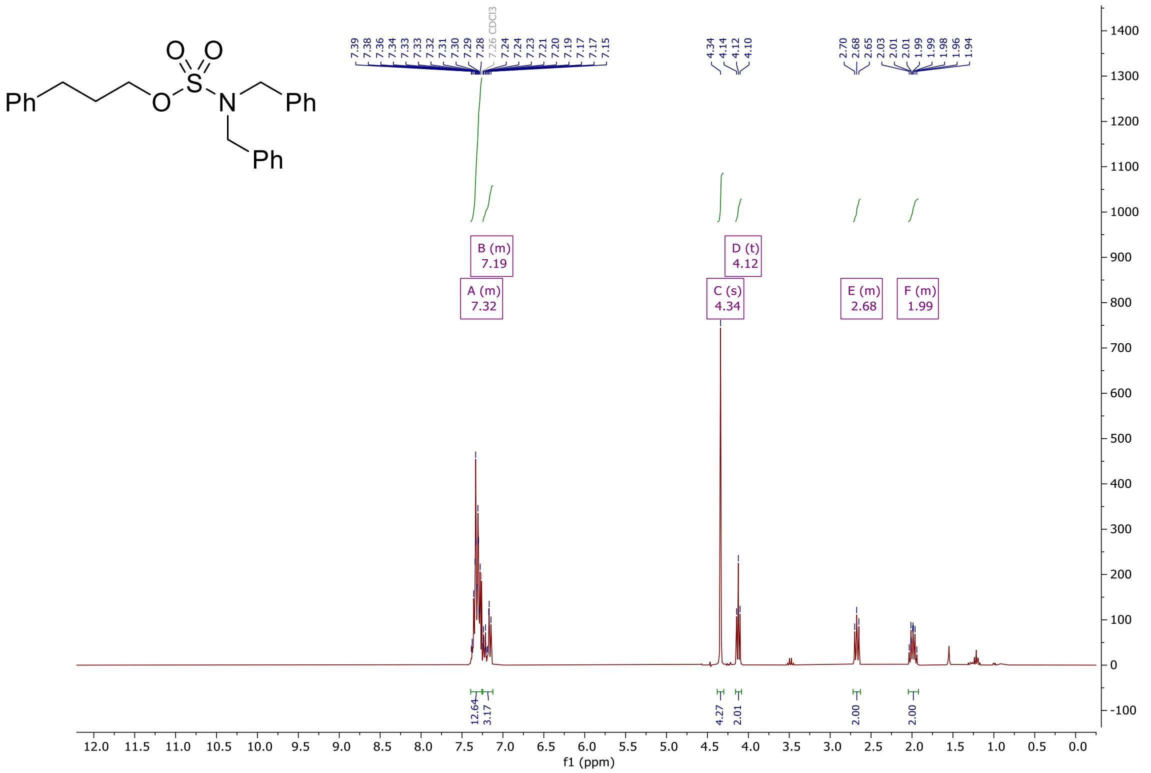

12e $^{13}C\{^{1}H\}$ NMR (75 MHz, Chloroform-*d*)

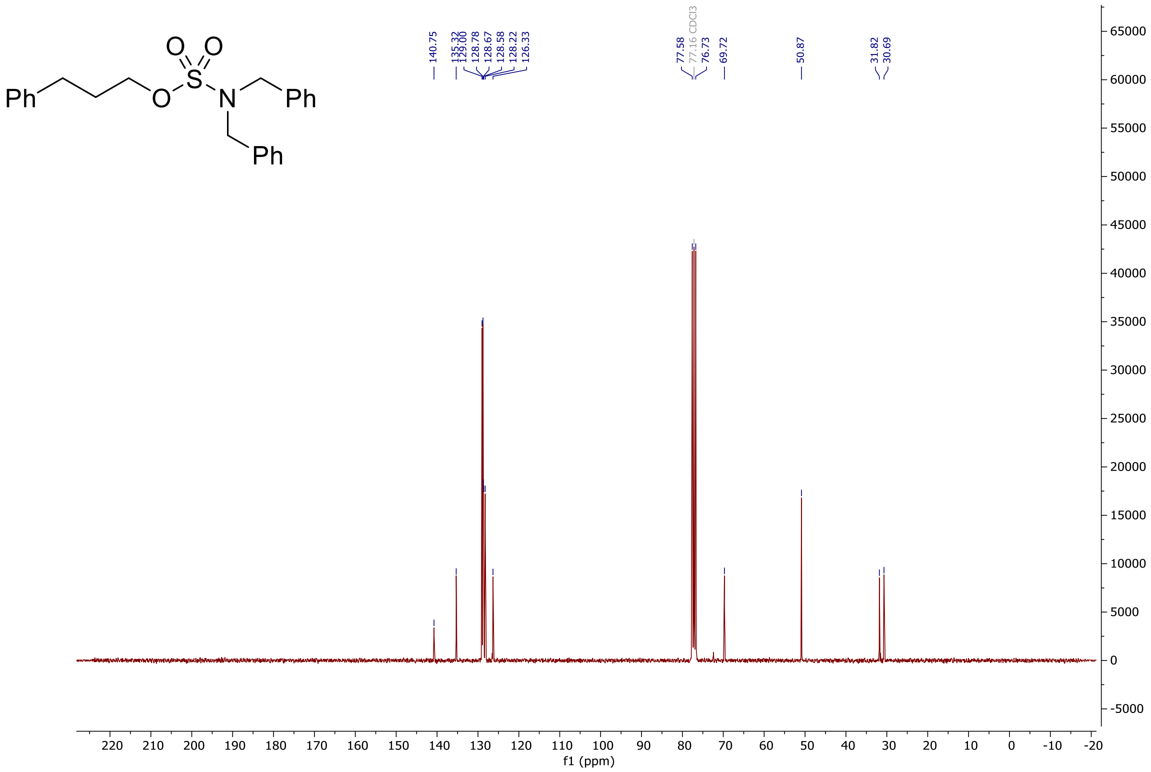

12f ^{1}H NMR (300 MHz, Chloroform-*d*)

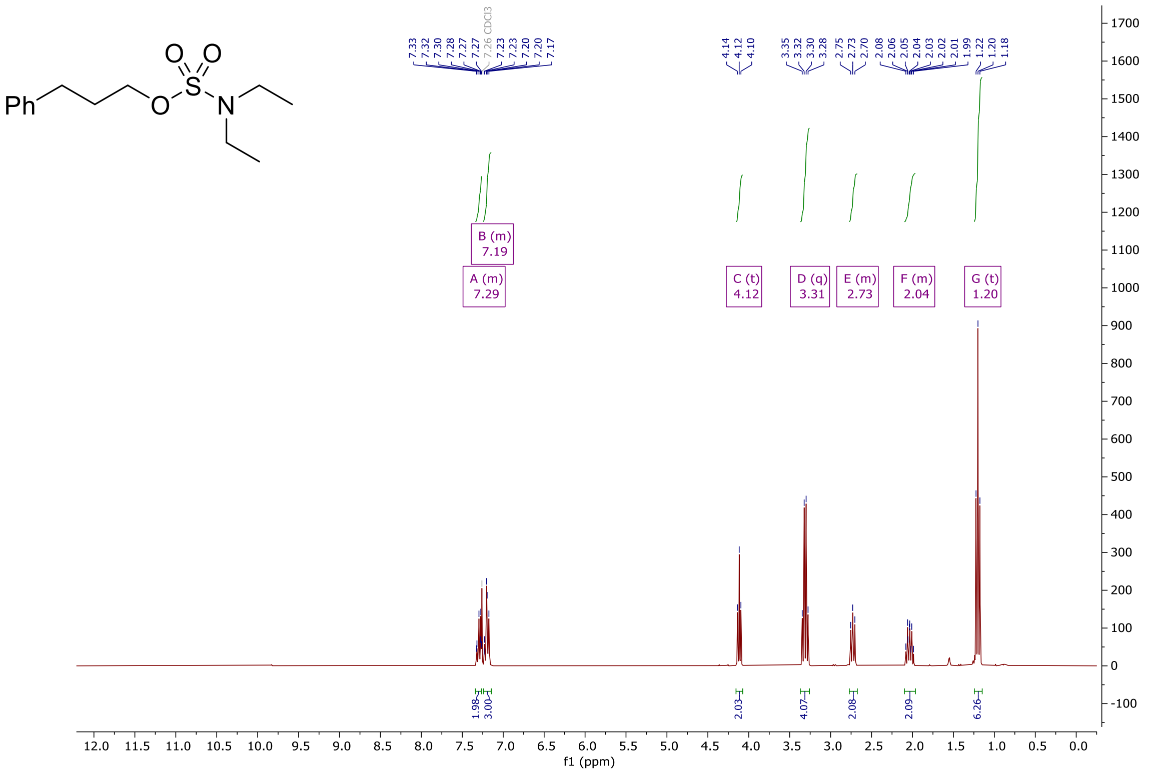

12f $^{13}C\{^{1}H\}$ NMR (75 MHz, Chloroform-*d*)

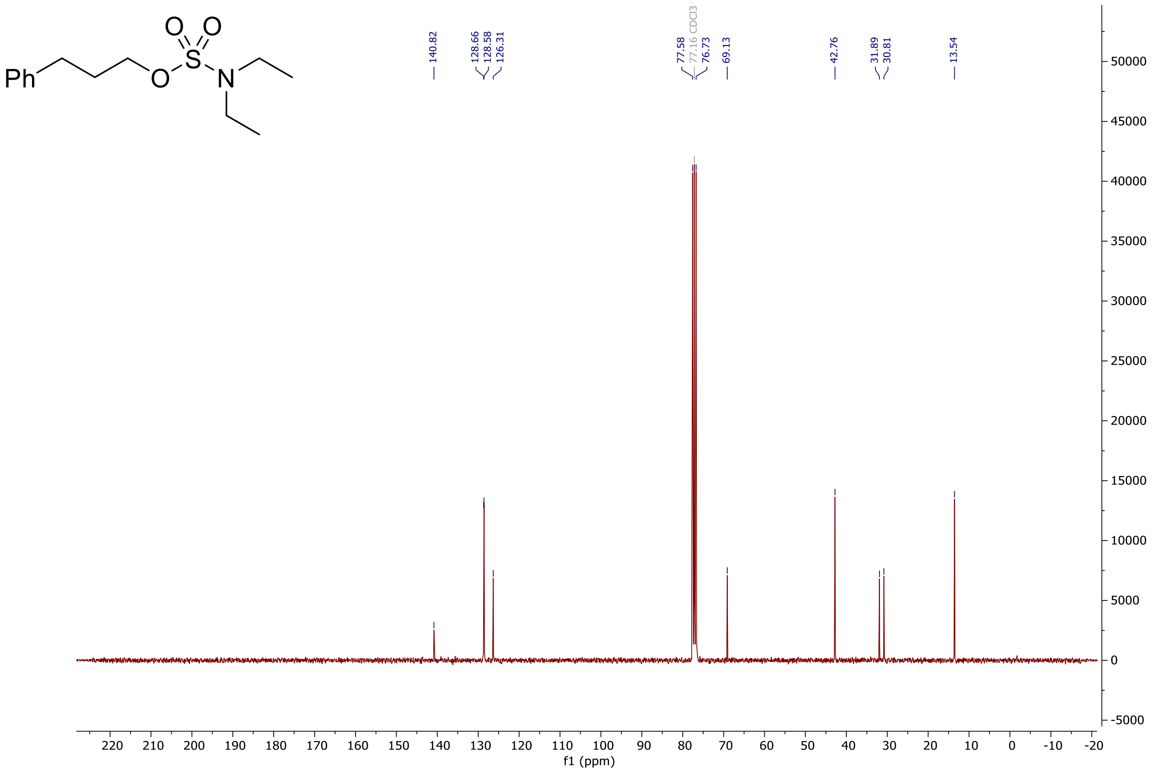

12g ^1H NMR (300 MHz, Chloroform-*d*)

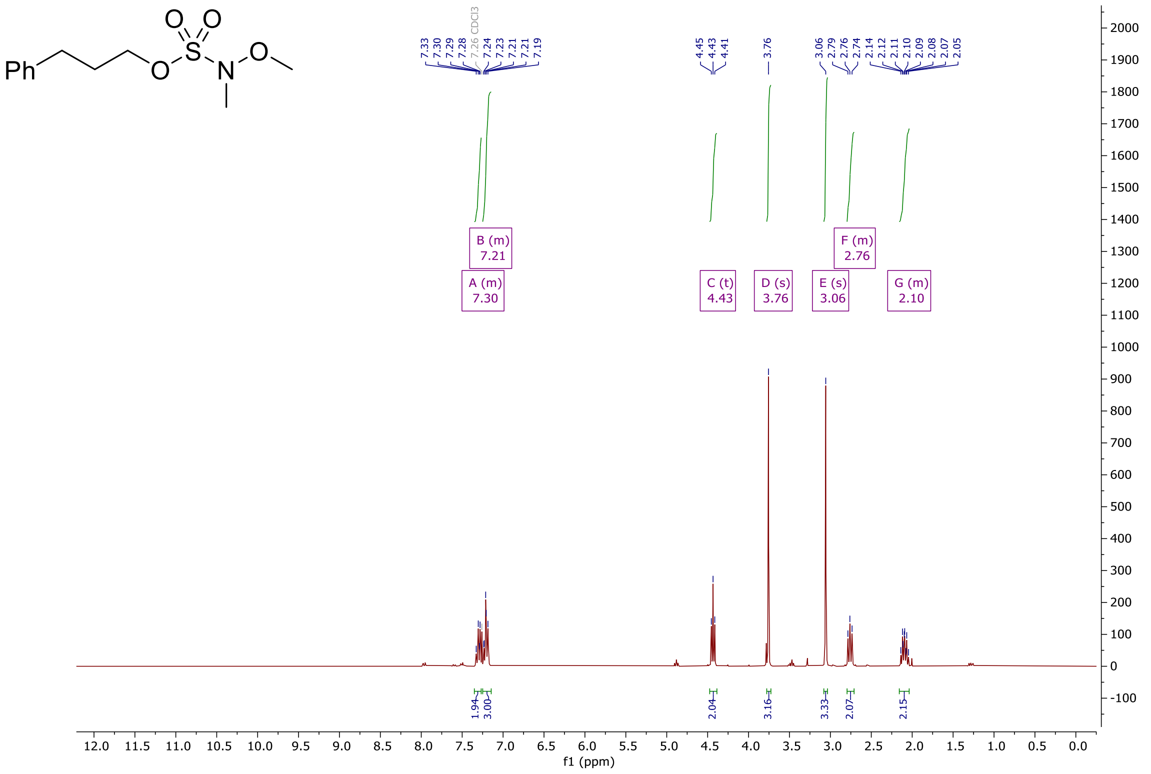

12g $^{13}C\{^1H\}$ NMR (75 MHz, Chloroform-*d*)

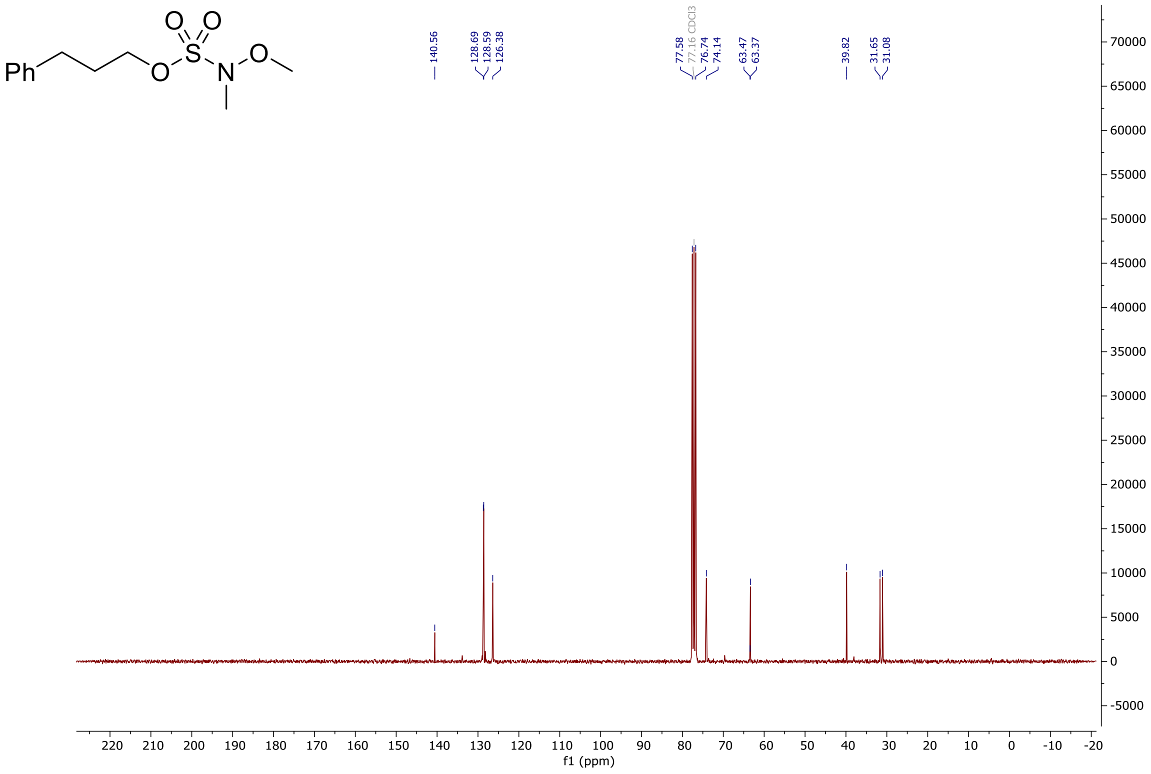

12h ^{1}H NMR (300 MHz, Chloroform-*d*)

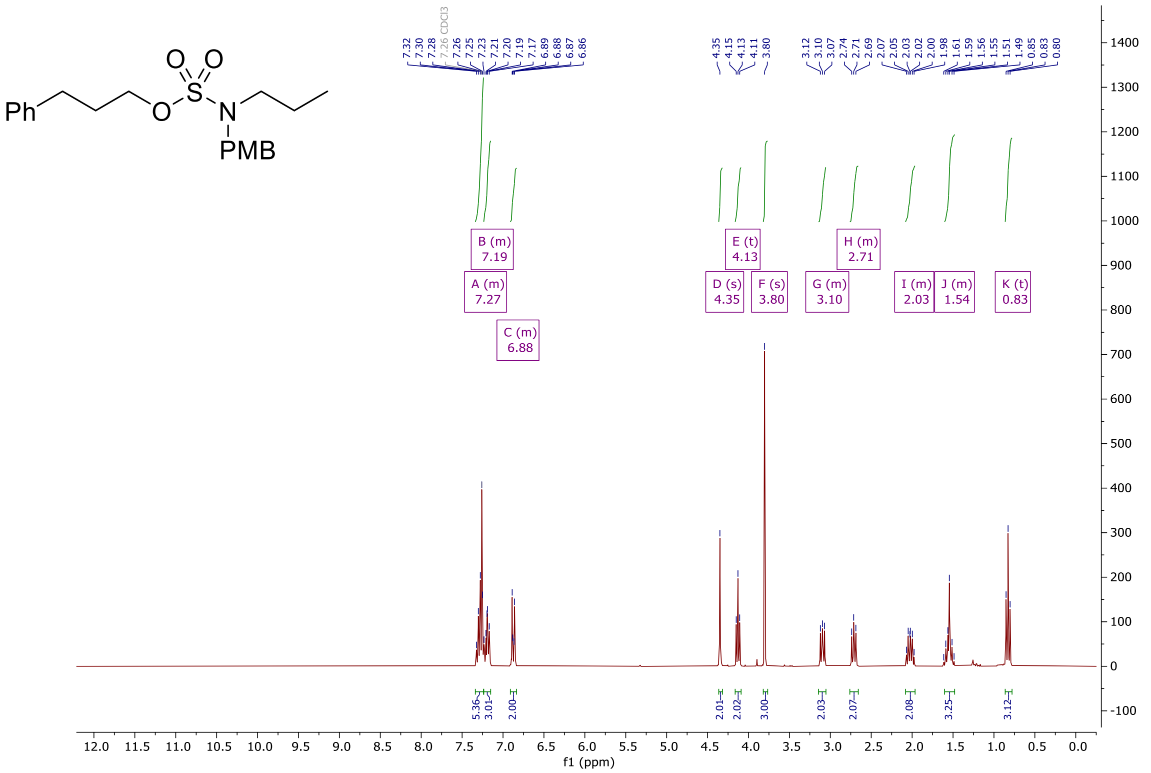

12h ^{13}C{^{1}H} NMR (75 MHz, Chloroform-*d*)

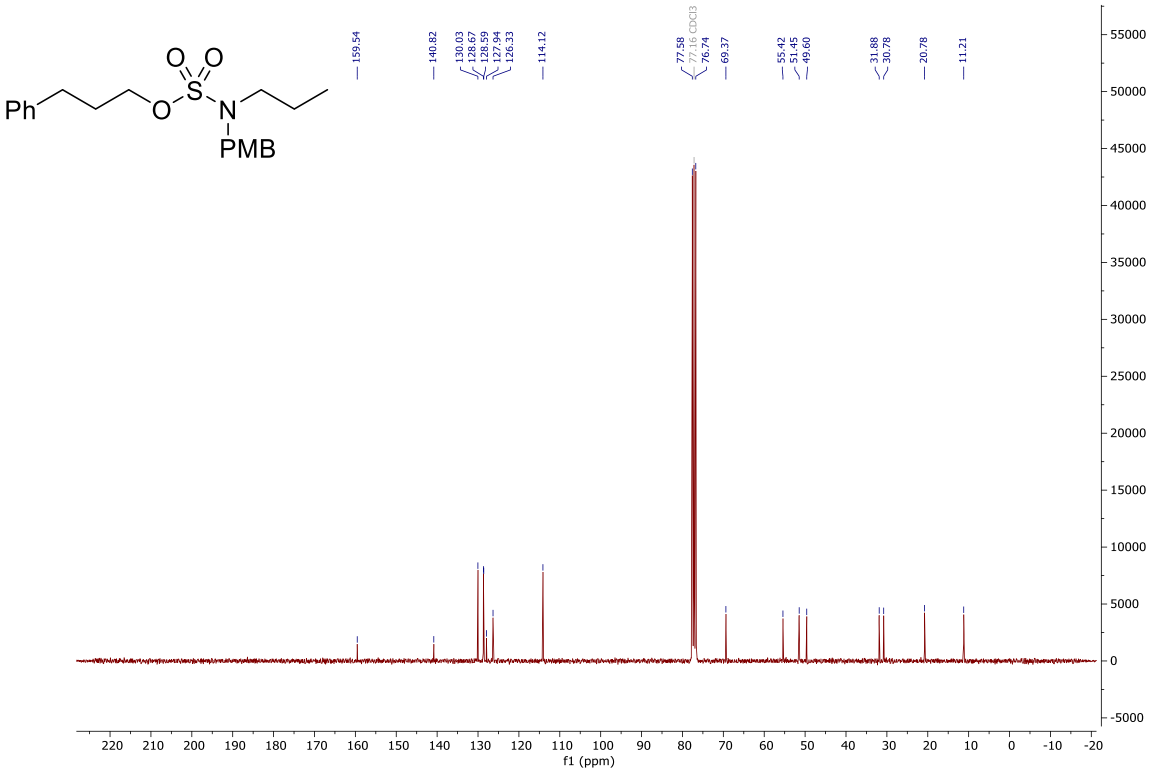

14 ^{1}H NMR (300 MHz, Chloroform-*d*)

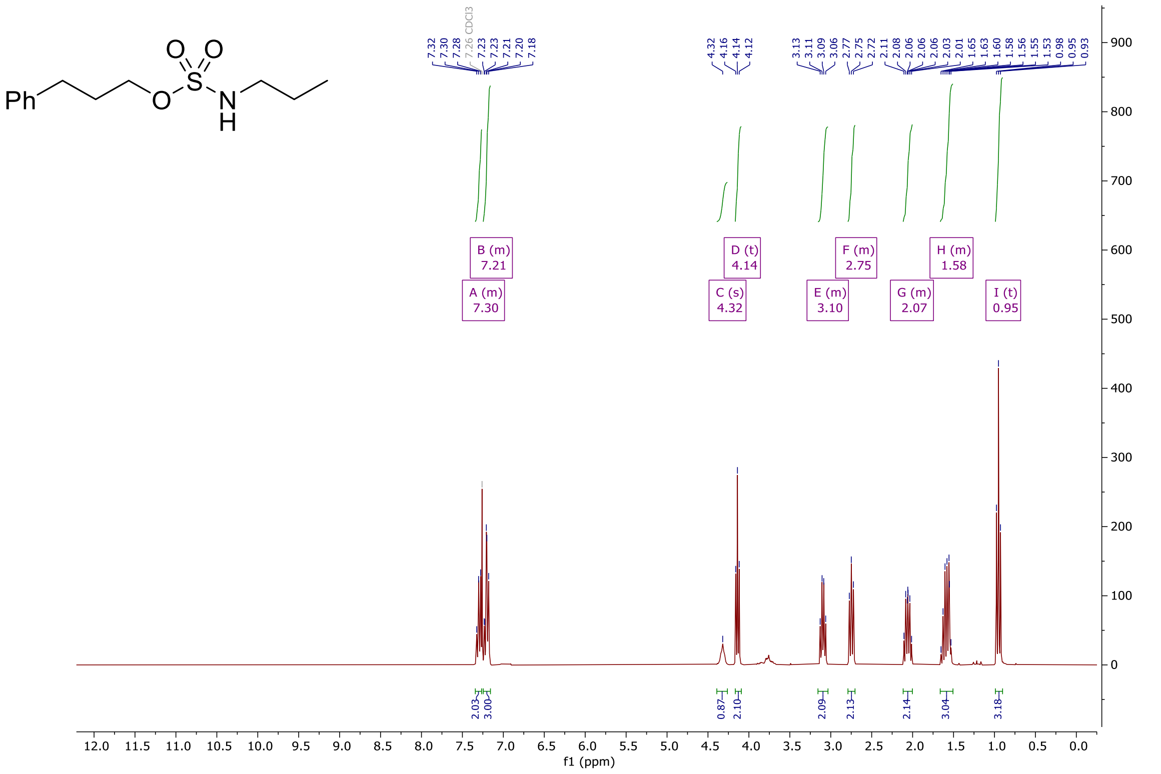

14 $^{13}C\{^{1}H\}$ NMR (75 MHz, Chloroform-*d*)

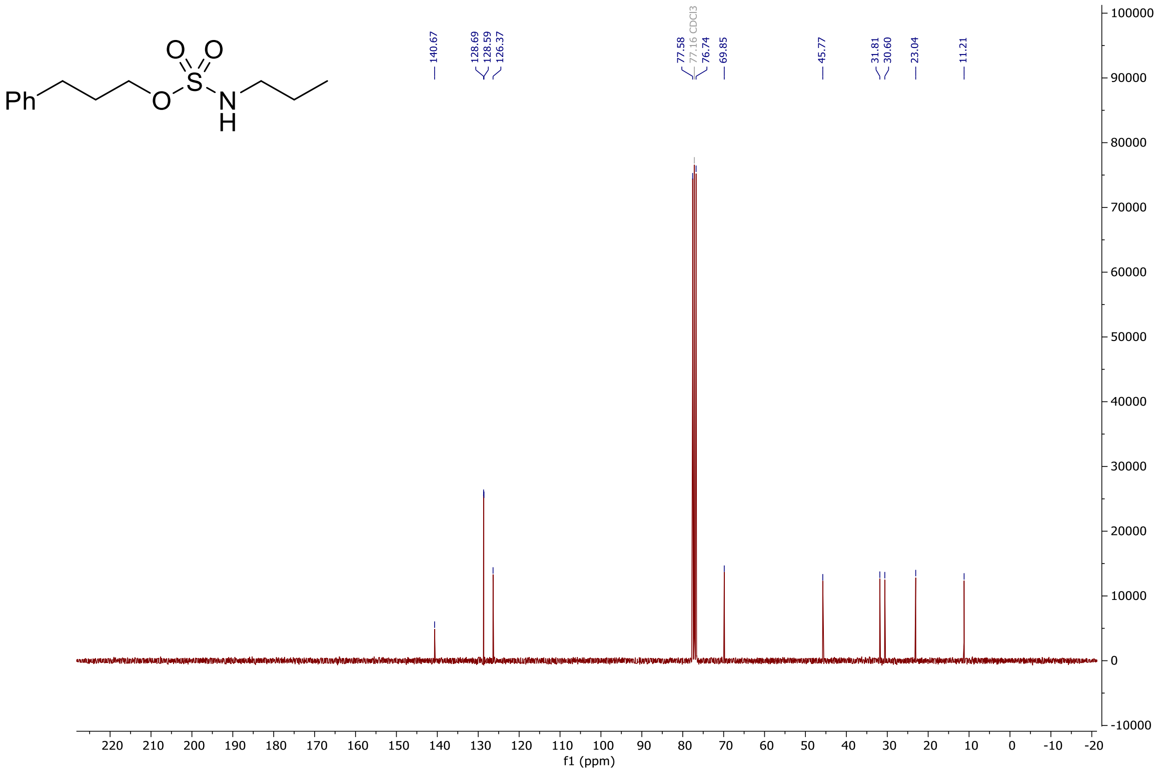

12i ^{1}H NMR (300 MHz, Chloroform-*d*)

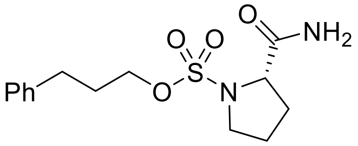

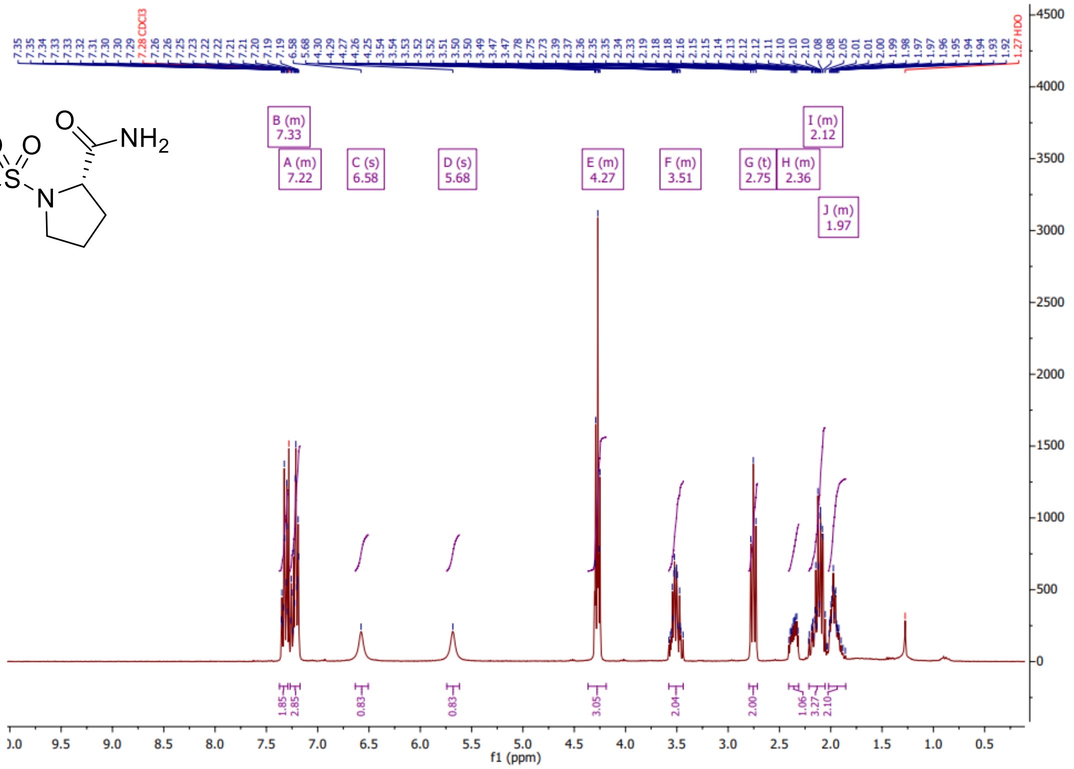

12i ^{13}C{^{1}H} NMR (75 MHz, Chloroform-*d*)

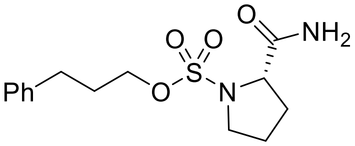

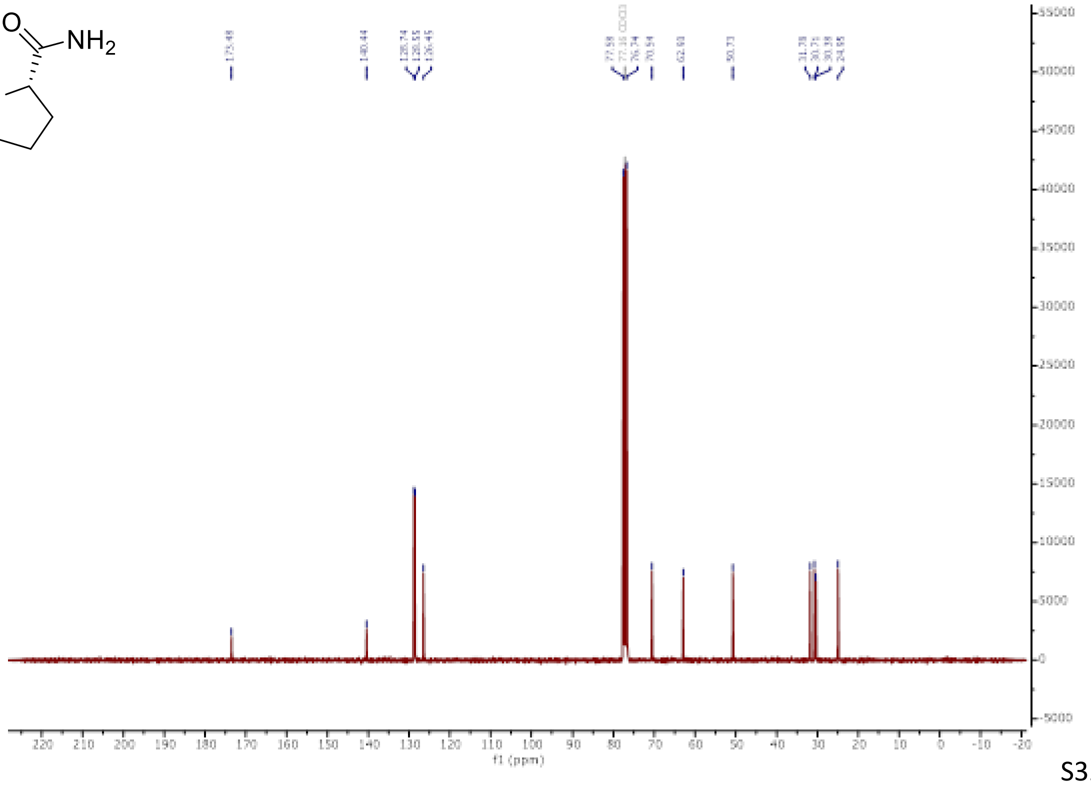

12j ¹H NMR (300 MHz, Chloroform-*d*)

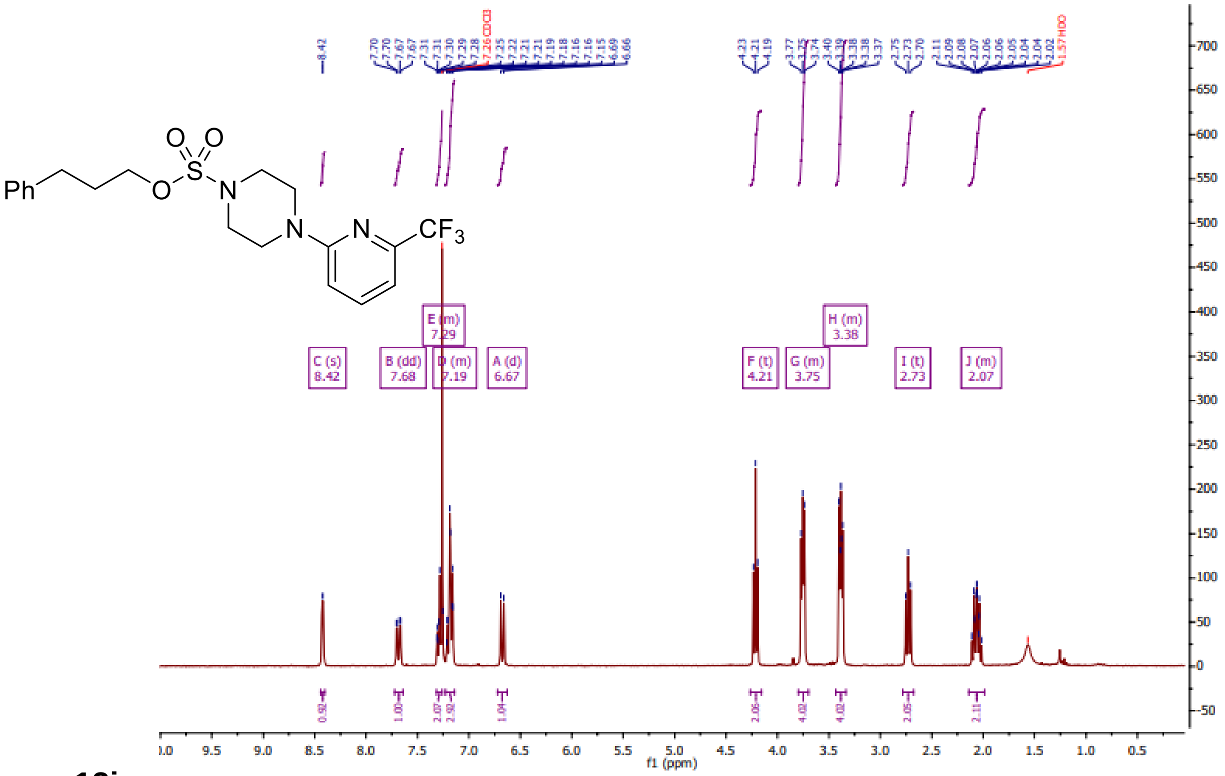

12j

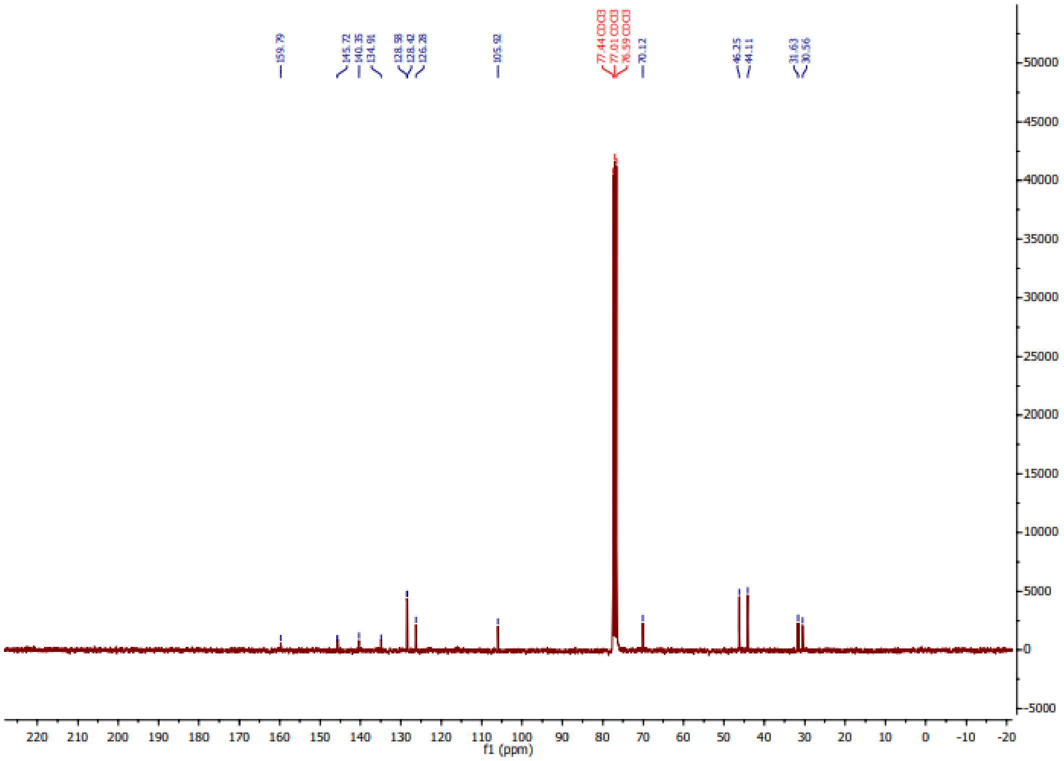

$^{13}C\{^{1}H\}$ NMR (75 MHz, Chloroform-*d*)

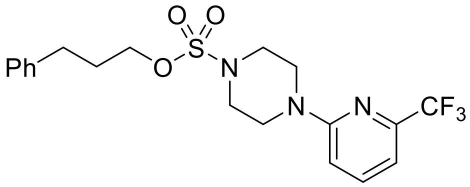

12r $^{19}F\{^{1}H\}$ NMR (282 MHz, Chloroform-*d*)

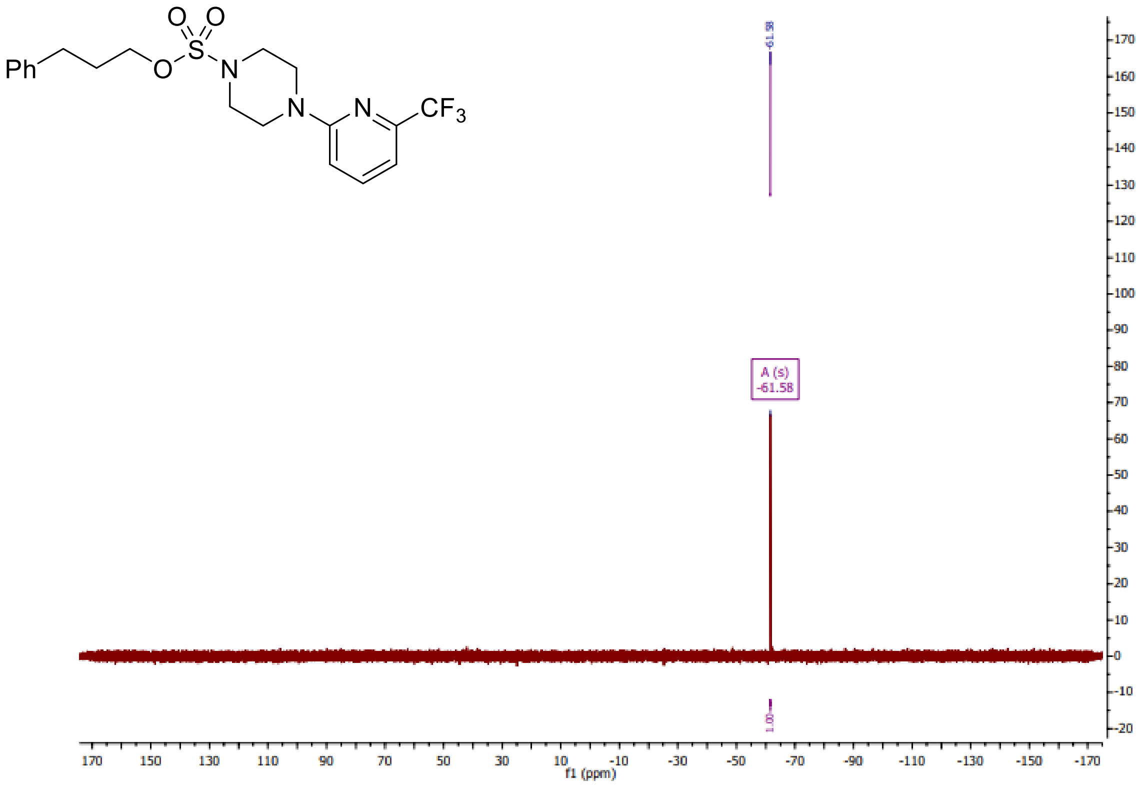

12k ^{1}H NMR (300 MHz, Chloroform-*d*)

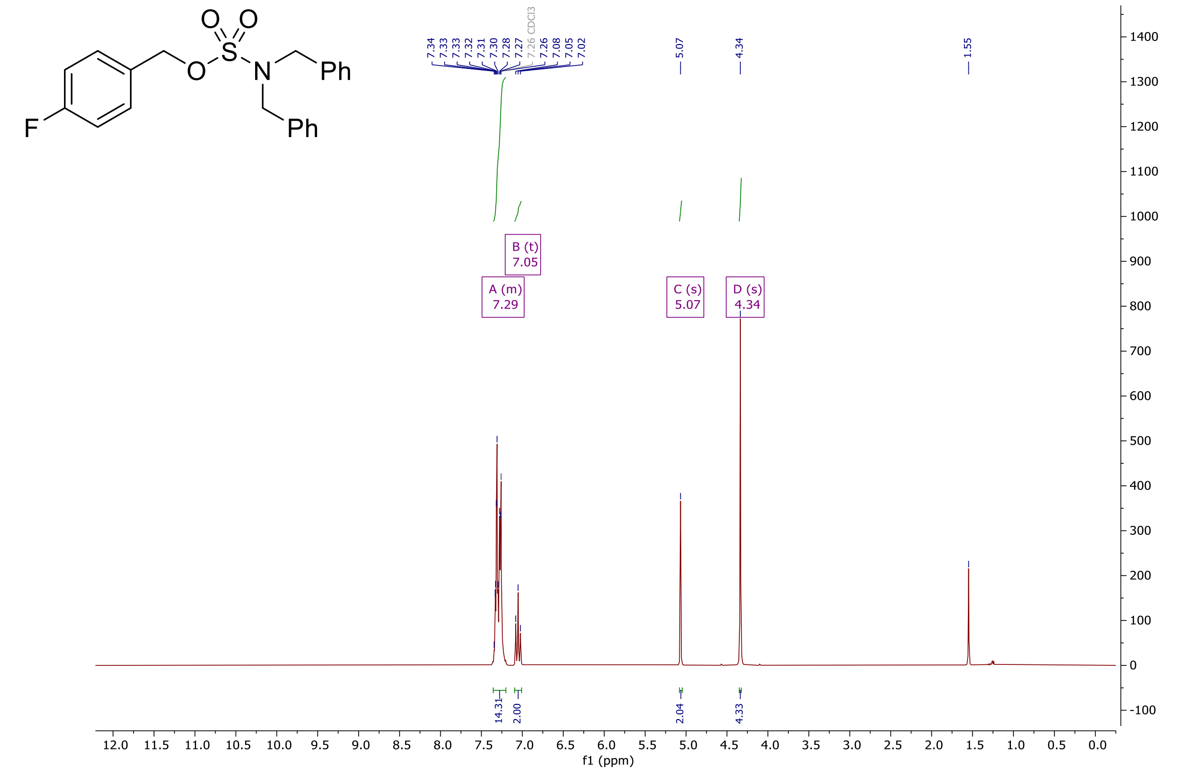

12k ^{13}C{^{1}H} NMR (75 MHz, Chloroform-*d*)

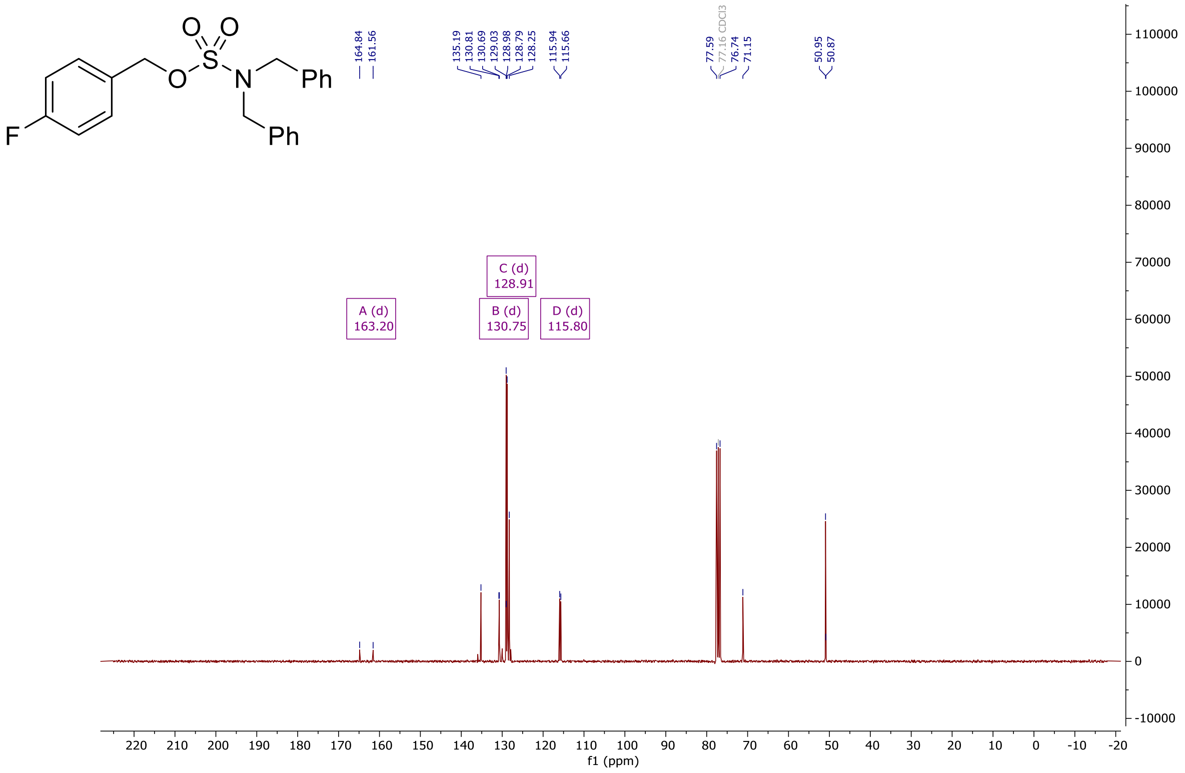

12k ^{19}F NMR (282 MHz, Chloroform-*d*)

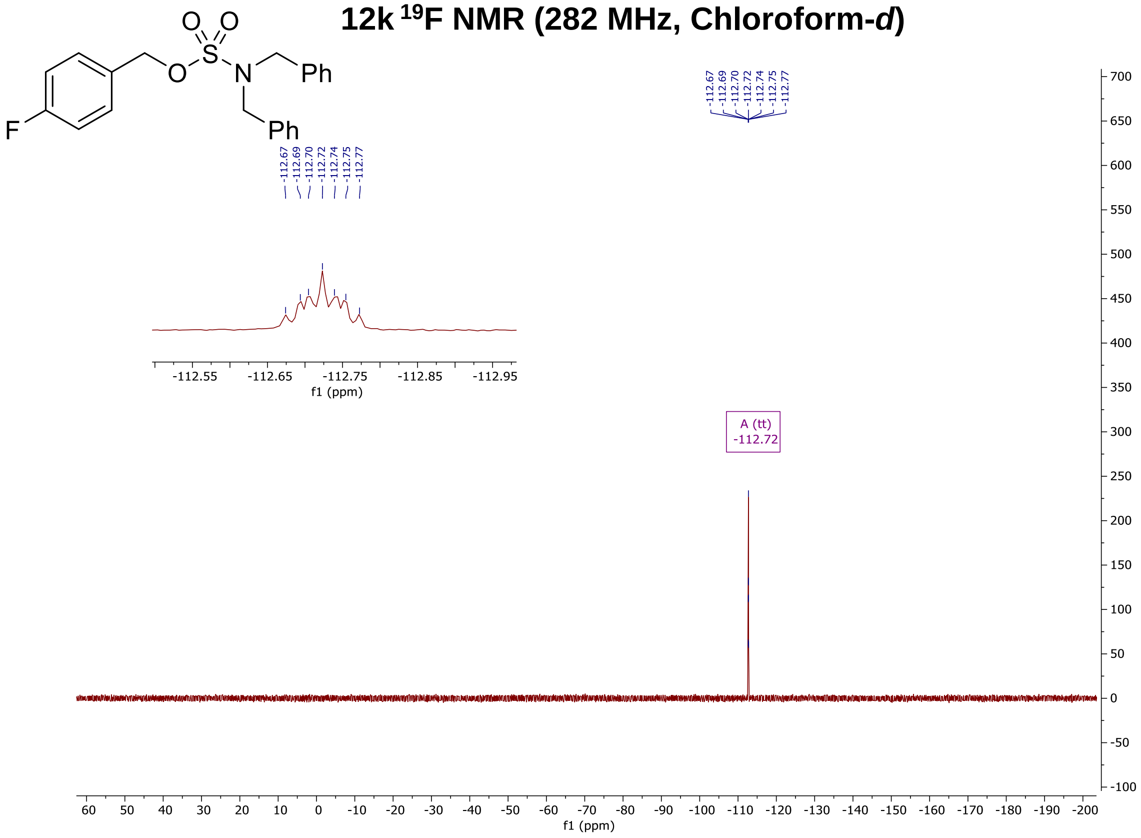

12l ^{1}H NMR (300 MHz, Chloroform-*d*)

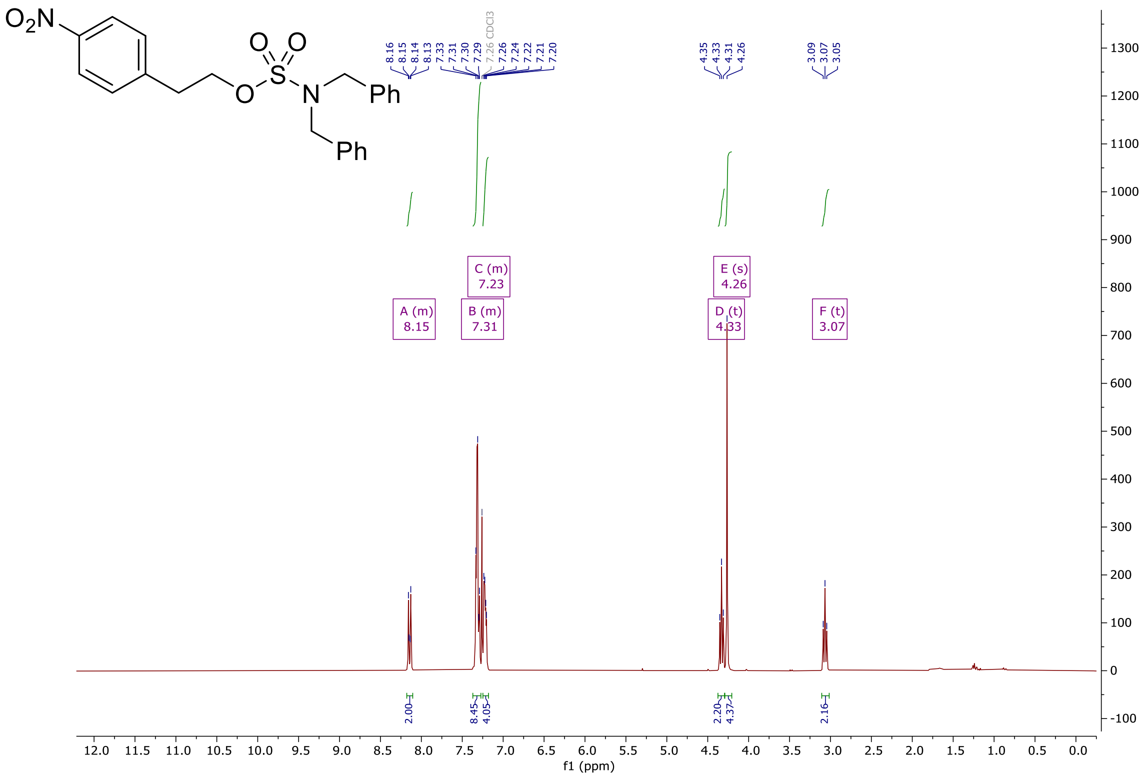

12l $^{13}C\{^{1}H\}$ NMR (75 MHz, Chloroform-*d*)

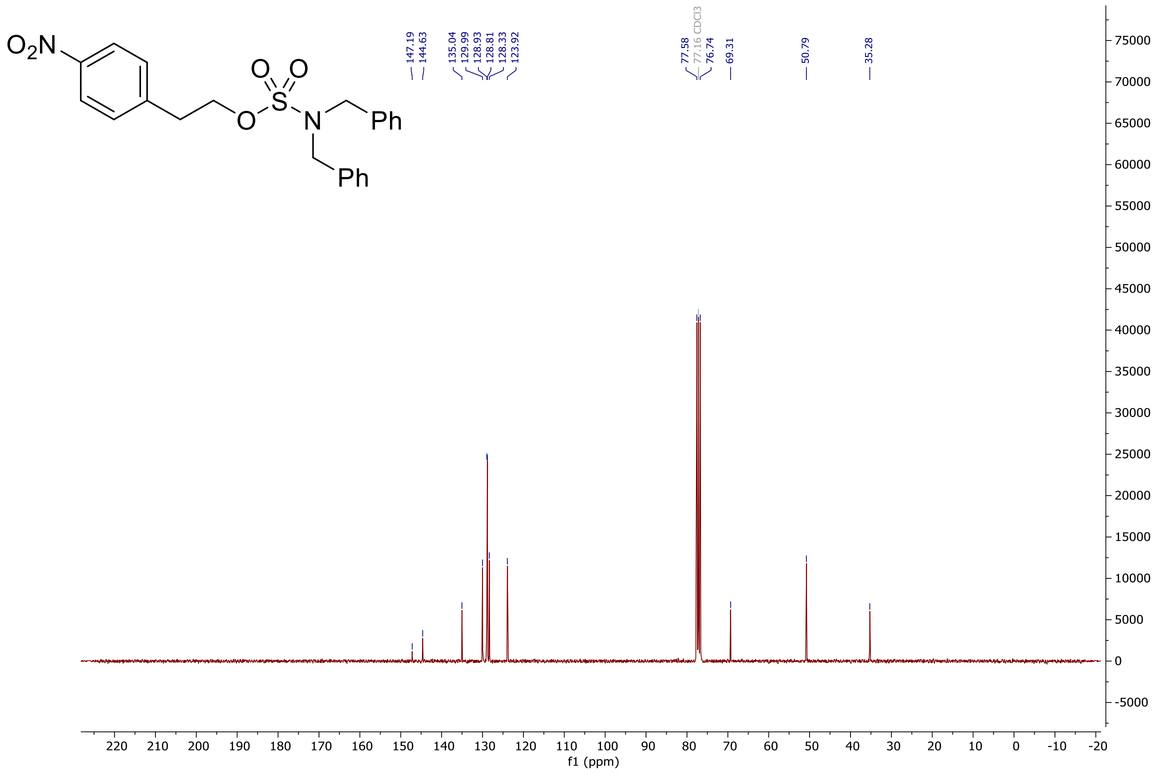

12m ^{1}H NMR (300 MHz, Chloroform-*d*)

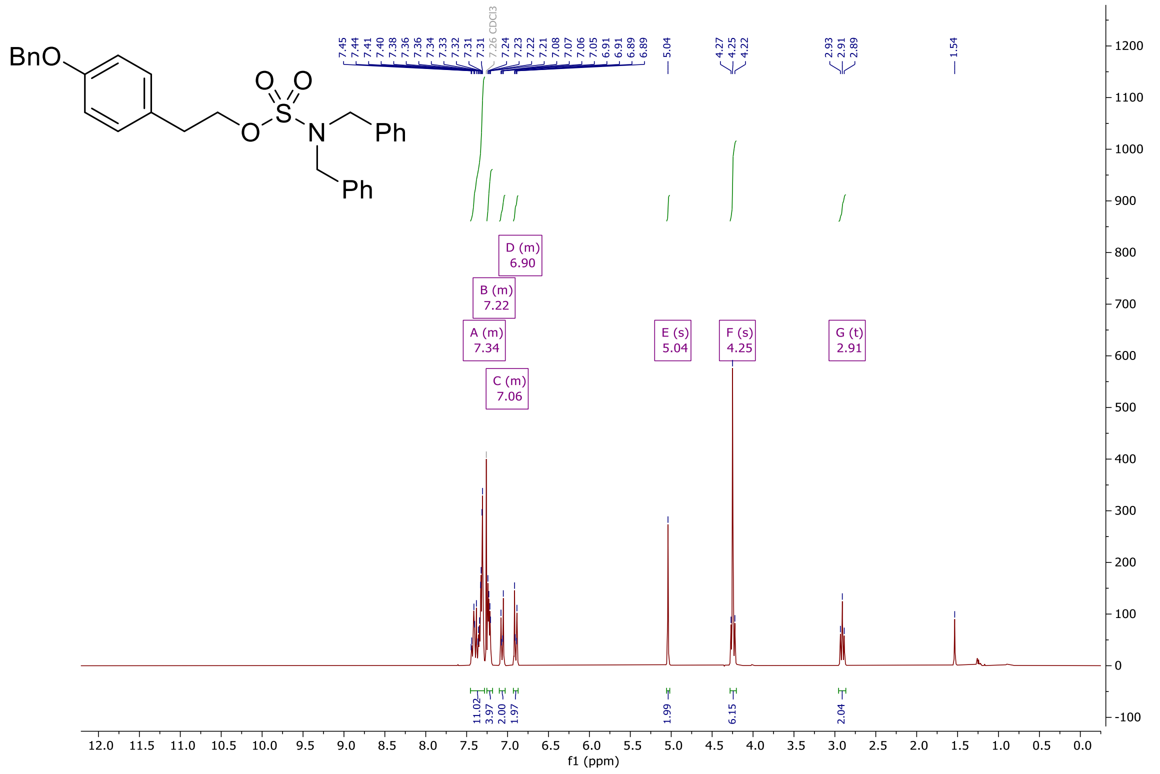

12m $^{13}C\{^{1}H\}$ NMR (75 MHz, Chloroform-*d*)

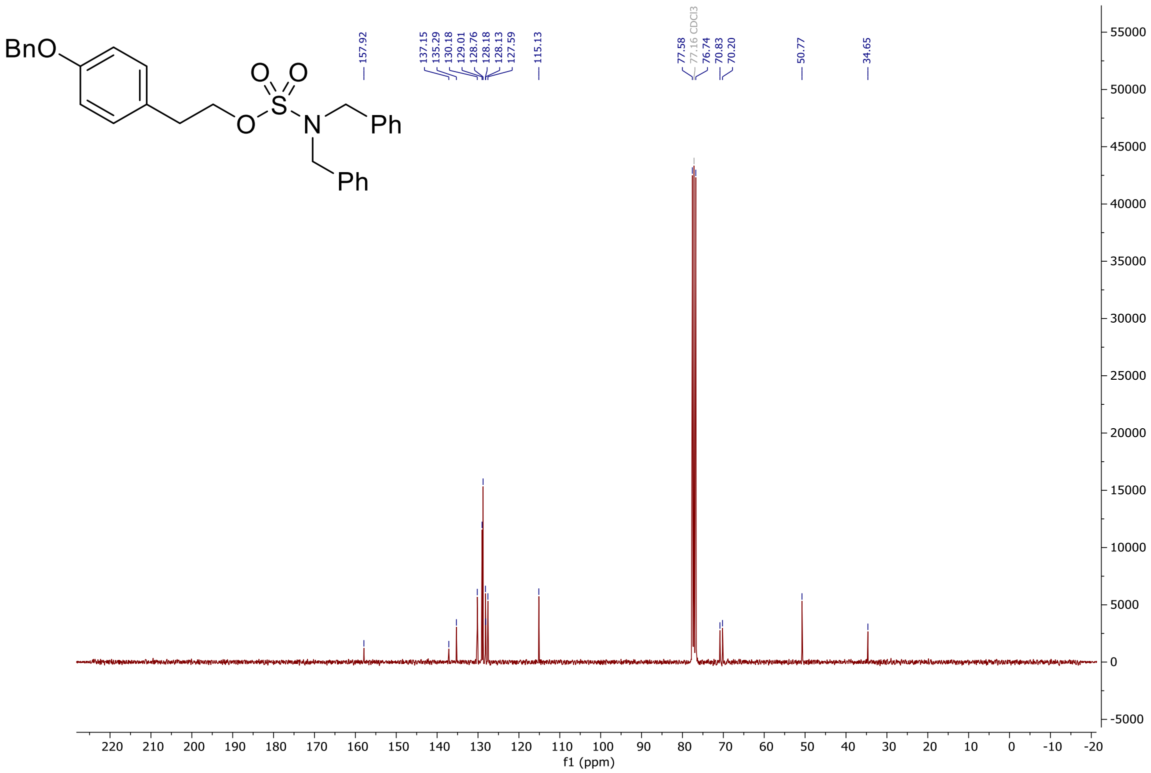

12n ^{1}H NMR (300 MHz, Chloroform-*d*)

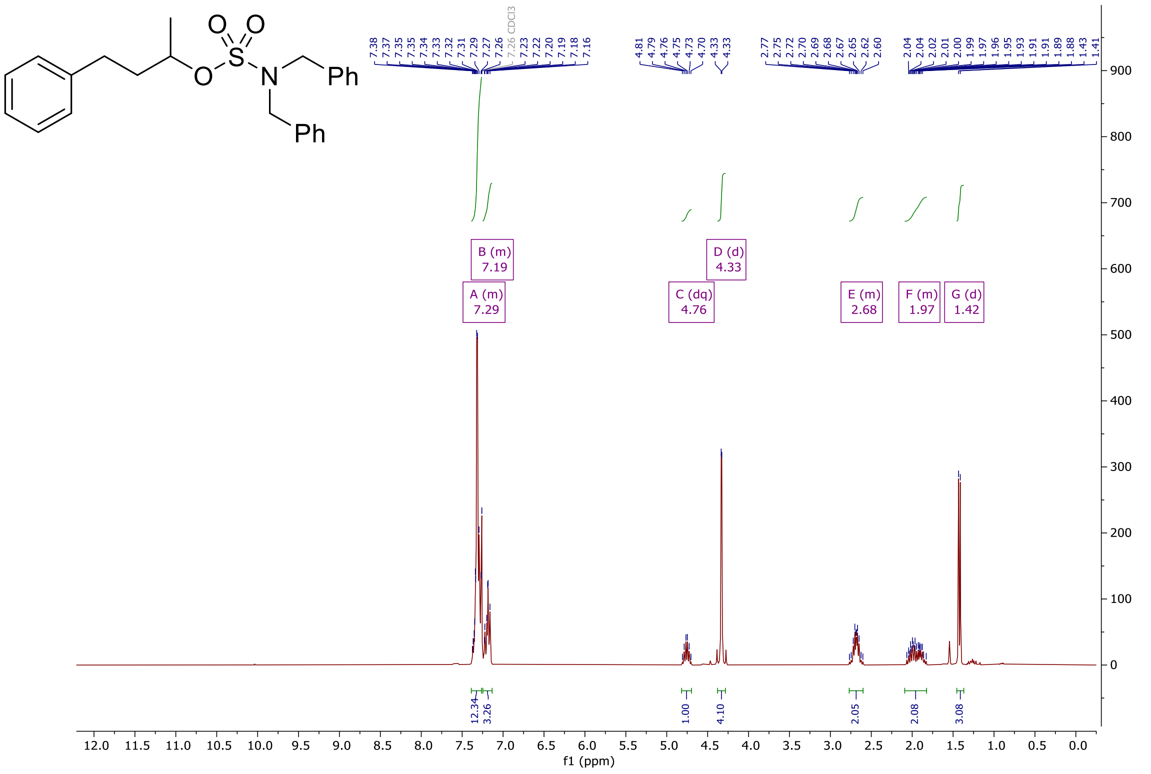

12n $^{13}C\{^{1}H\}$ NMR (75 MHz, Chloroform-*d*)

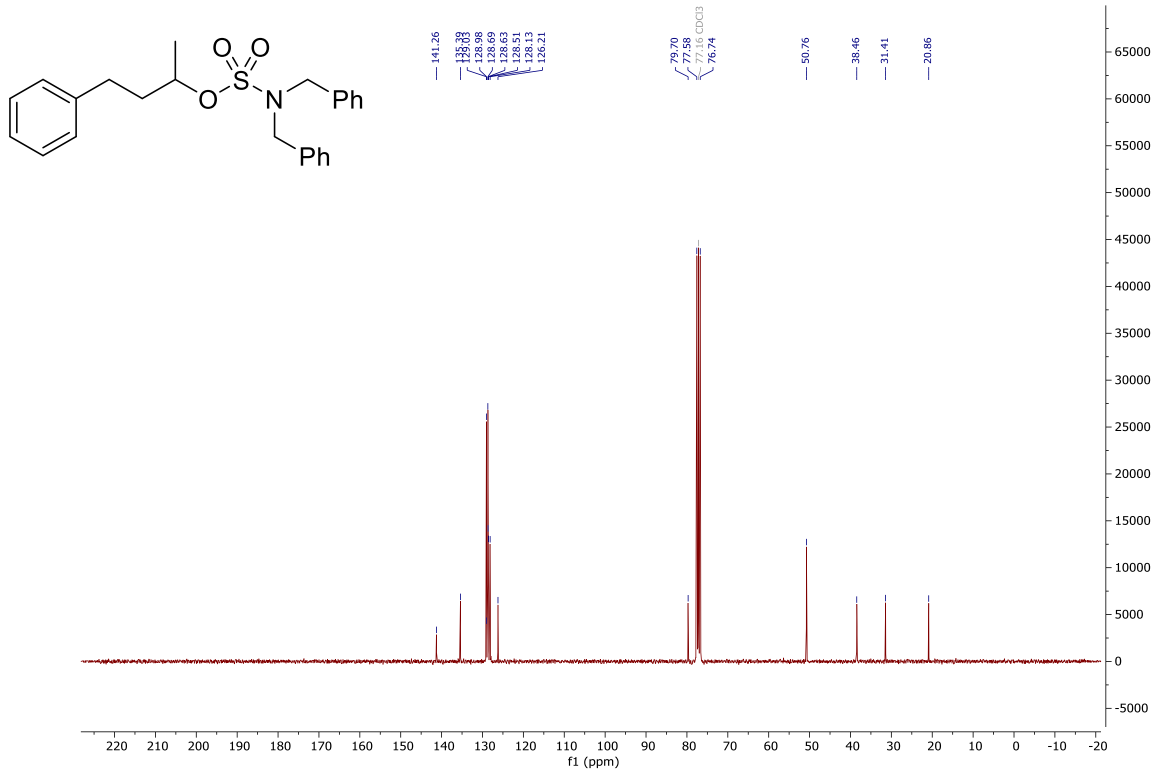

12o ^{1}H NMR (300 MHz, Chloroform-*d*)

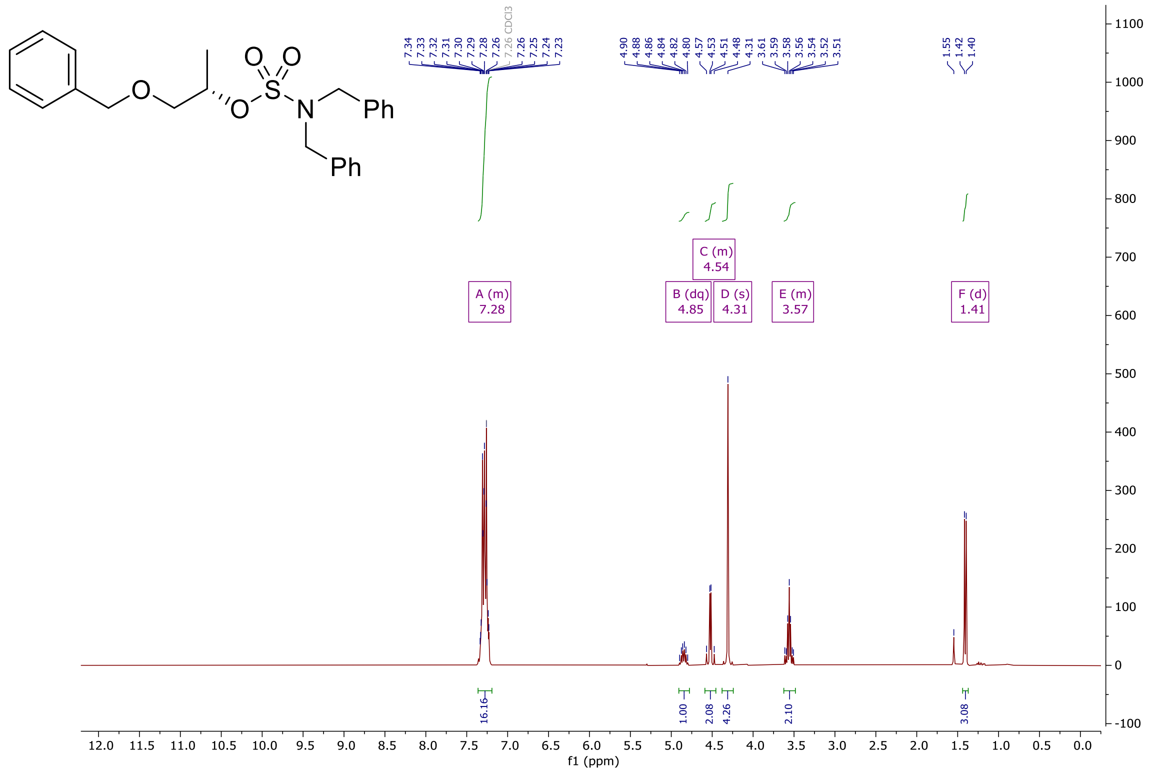

12o $^{13}C\{^1H\}$ NMR (75 MHz, Chloroform-*d*)

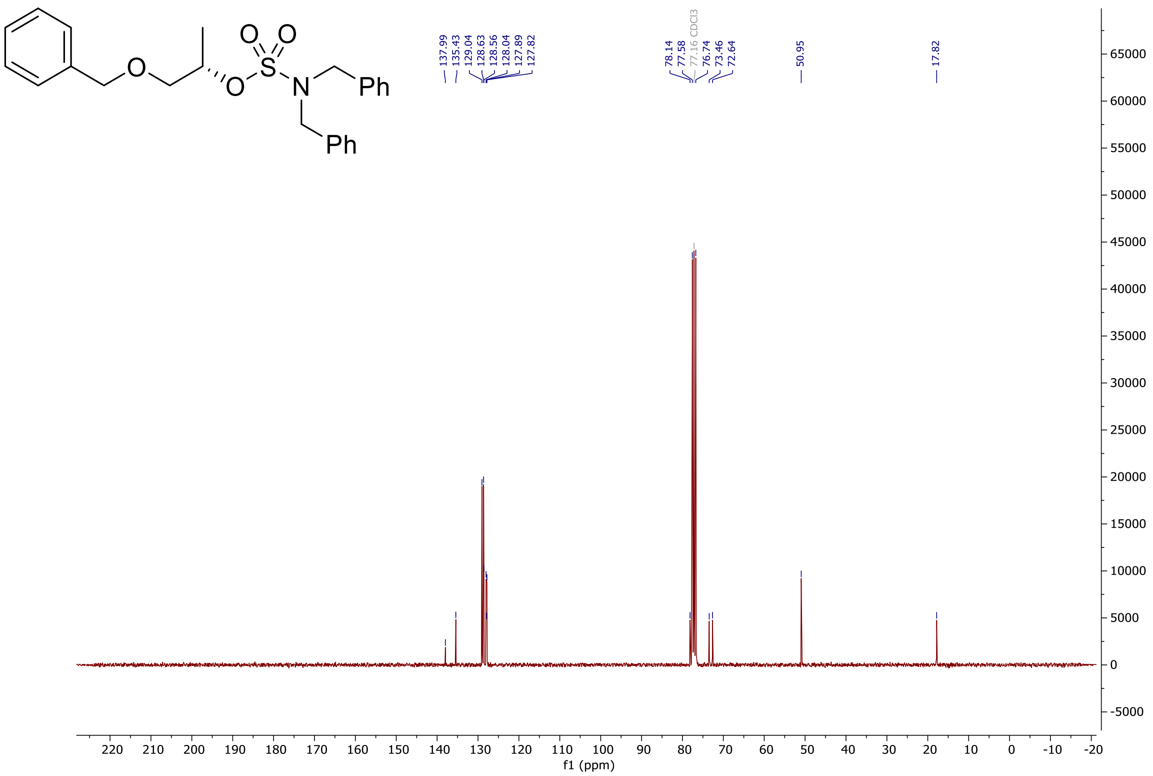

12p ^1H NMR (300 MHz, Chloroform-*d*)

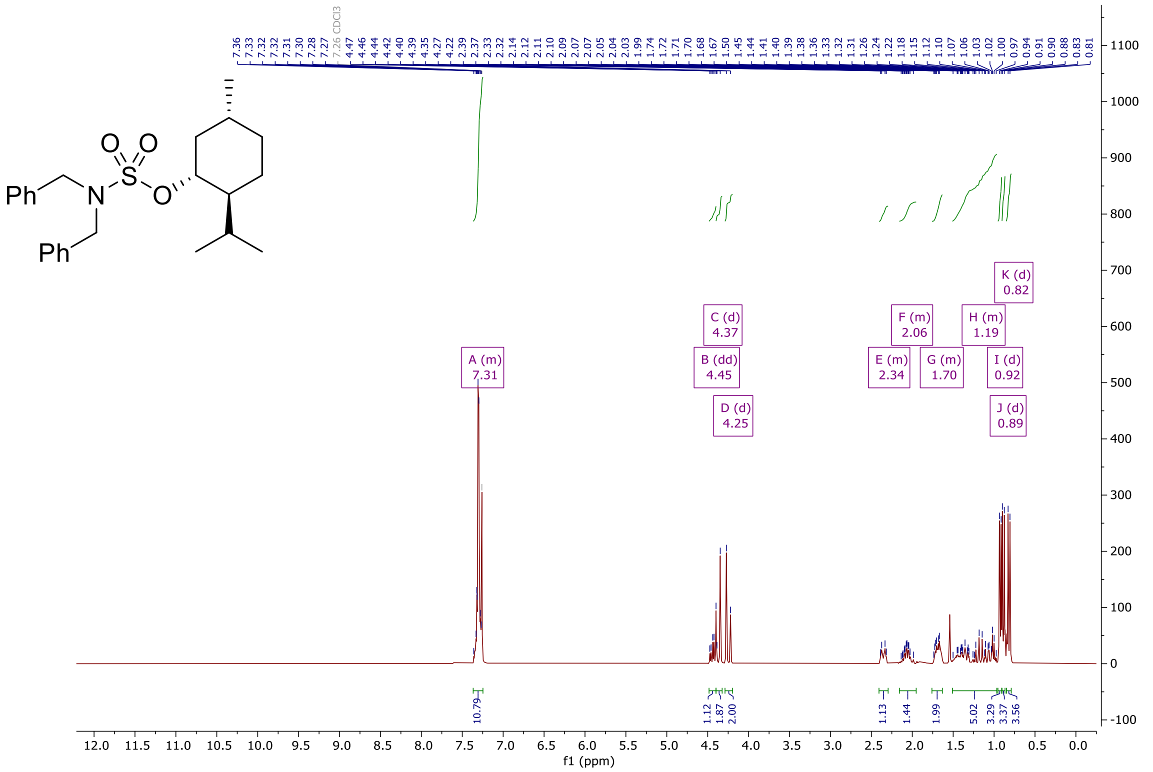

12p $^{13}C\{^1H\}$ NMR (75 MHz, Chloroform-*d*)

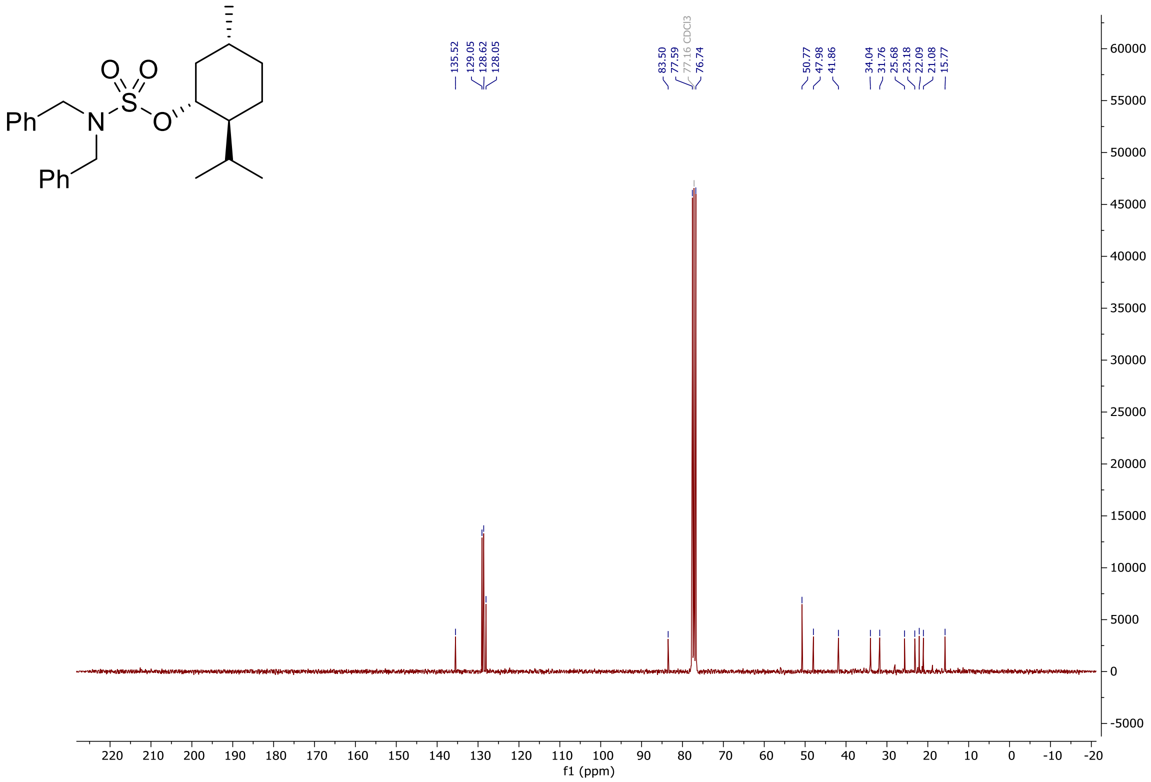

12q ^1H NMR (300 MHz, Chloroform-*d*)

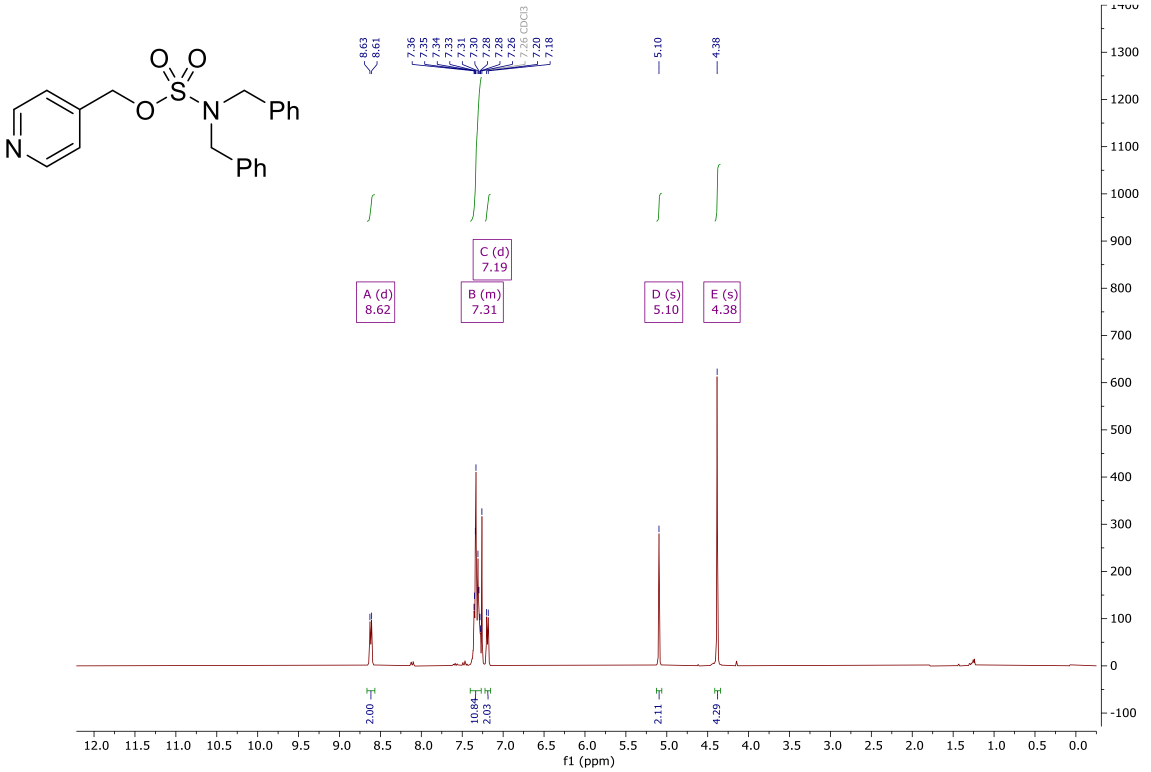

12q $^{13}C\{^{1}H\}$ NMR (75 MHz, Chloroform-*d*)

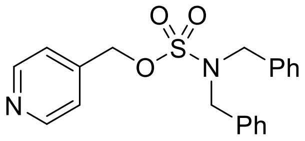

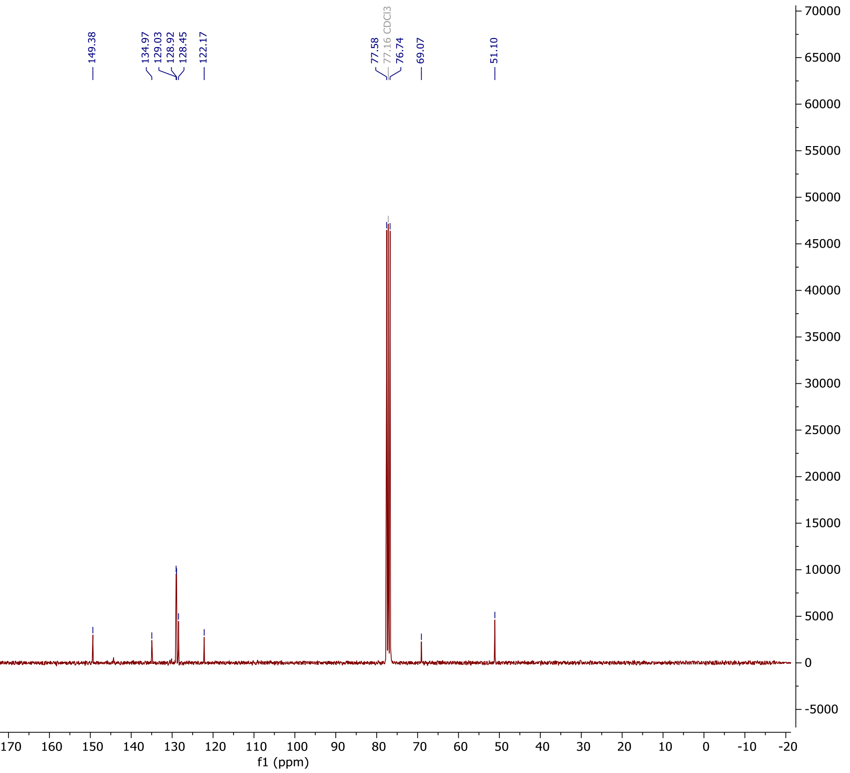

12r ^{1}H NMR (300 MHz, Chloroform-*d*)

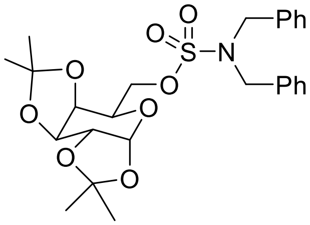

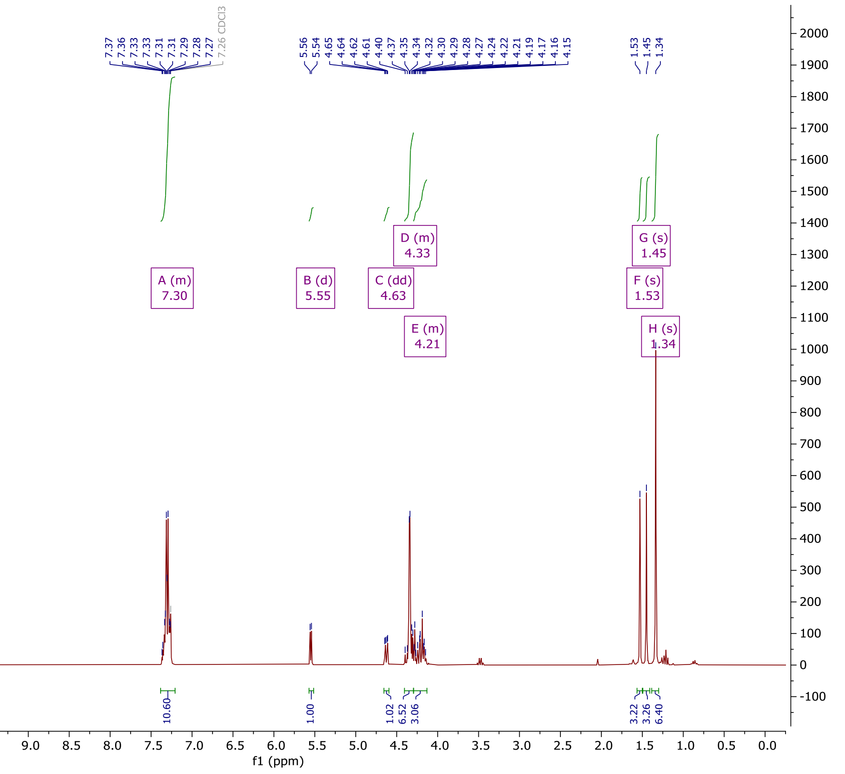

12r $^{13}C\{^{1}H\}$ NMR (75 MHz, Chloroform-*d*)

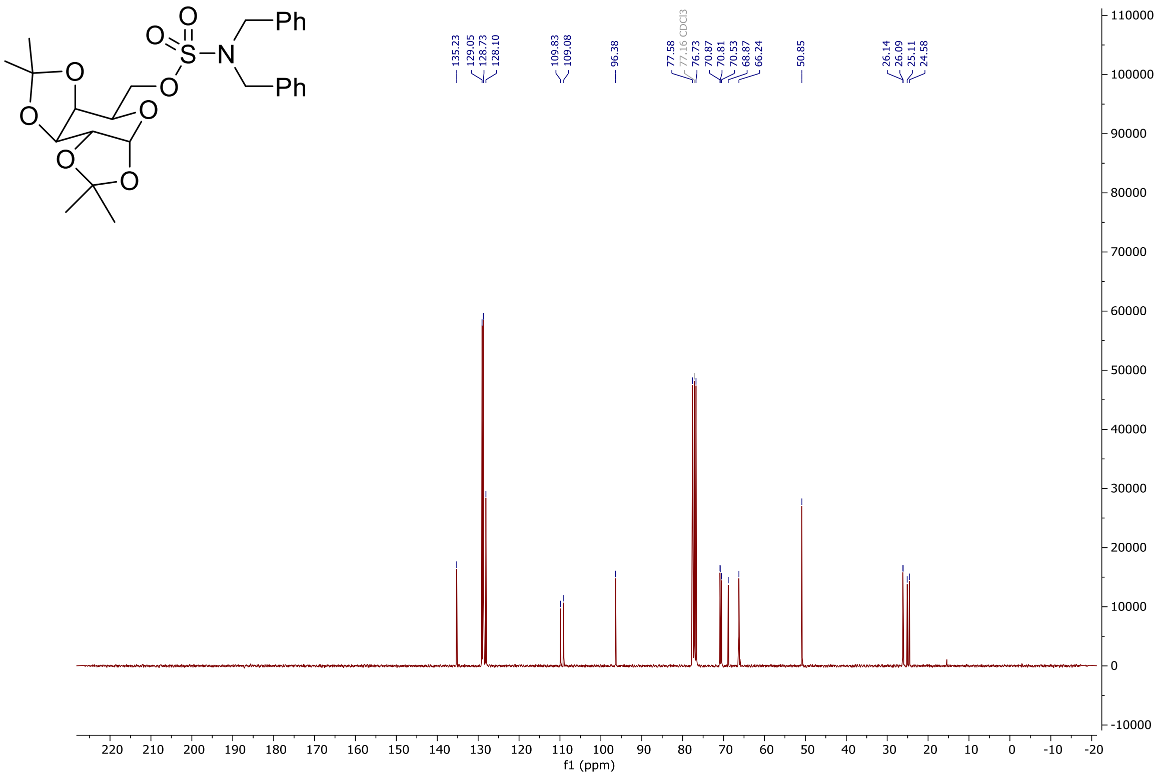

Supplement: SC-014-D2SC05945B-s001 [file SC-014-D2SC05945B-s001.pdf]
